# Integration of Transcriptomics and Metabolomics Reveals the Antitumor Mechanism of Protopanaxadiol Triphenylphosphate Derivative in Non-Small-Cell Lung Cancer

**DOI:** 10.3390/molecules29174275

**Published:** 2024-09-09

**Authors:** Liu Han, Xingbo Bian, Xiangyu Ma, Ting Ren, Yawei Li, Lijing Huang, Zebo Tang, Liancong Gao, Sheng Chang, Xin Sun

**Affiliations:** 1 College of Pharmacy, Jilin Medical University, Jilin 132013, China; hanliu@jlmu.cn (L.H.); bxb4532@163.com (X.B.); y927597907@163.com (X.M.); rentingvip@163.com (T.R.); lyw135@163.com (Y.L.); 13018538662@163.com (L.H.); 2 School of Basic Medicine, Jilin Medical University, Jilin 132013, China; tzbking122981@163.com; 3 Clinical Medical School, Jilin Medical University, Jilin 132013, China; gaolc@jlmu.cn

**Keywords:** protopanaxadiol triphenylphosphate derivative, non-small-cell lung cancer (NSCLC), antitumor mechanism, transcriptome, metabonomics

## Abstract

The objective of this study was to enhance the membrane permeability and anticancer effectiveness of (20*S*)-protopanaxadiol (PPD) by introducing triphenylphosphonium into the OH group at the C-3 site. This study shows that the anti-proliferation activity of CTPPPPD, with an IC50 value of 1.65 ± 0.10 μmol/L, was 33-times better than that of PPD (with an IC50 value of 54.56 ± 4.56 μmol/L) and superior to that of cisplatin (with an IC50 value of 1.82 ± 0.25 μmol/L) against A549 cells. Biological examinations suggested that CTPPPPD treatment reduced the growth rate of A549 cells, increased the permeability of cell membranes, and changed the structure of chromosomal DNA in a concentration-dependent manner. Annexin V/PI assay and flow cytometry were employed to detect the effect of CTPPPPD on the apoptosis of A549 cells. The results showed that CTPPPPD could induce the apoptosis of A549 cells, and the apoptosis rate of A549 cells treated with 0, 1.0, 2.0, and 4.0 μM of CTPPPPD for 24 h was 0%, 4.9%, 12.7%, and 31.0%, respectively. The integration of transcriptomics and metabolomics provided a systematic and detailed perspective on the induced antitumor mechanisms. A combined analysis of DEGs and DAMs suggested that they were primarily involved in the central carbon metabolism pathway in cancer, as well as the metabolism of aminoacyl-tRNA biosynthesis, alanine, aspartate, and glutamate. Central carbon metabolism in cancer-related genes, i.e., SLC16A3, FGFR3, LDHA, PGAM1, and SLC2A1, significantly reduced after treatment with CTPPPPD. In particular, the dominant mechanism responsible for total antitumor activity may be attributed to perturbations in the PI3K-AKT, MAPK, and P53 pathways. The findings derived from transcriptomics and metabolomics were empirically confirmed through q-PCR and molecular docking. Further analyses revealed that CTPPPPD could be a promising lead for the development of protopanaxadiol for non-small-cell lung cancer (NSCLC) drugs.

## 1. Introduction

Lung cancer originates from the mucous membranes of the lung glands or bronchi and is a major cause of cancer-related mortalities worldwide [1]. It has turned into a global public health concern due to the annual increase in its incidence and mortality over the past few decades [2]. The prevention and treatment of lung cancer have received international attention. NSCLC comprises around 85–90% of total lung cancer cases [3]. Several pathogenic mechanisms of lung cancer, including ALK fusion and EGFR mutations, have been identified, and novel therapeutic approaches, such as targeted therapy and immunotherapy, have been developed in recent years; however, the efficacy of early diagnosis and treatment for NSCLC is still unsatisfactory [4]. Currently, surgery is the primary treatment option for NSCLC with a 5-year survival rate of approximately 15–20% [5]. The therapeutic outcomes for NSCLC patients depend on the growth, movement, and infiltration of cancerous cells, especially the control of essential genes at the transcriptional level. Hence, it is necessary to identify new therapeutic agents that can target these crucial genes to enhance the efficacy of treatments.

Mitochondria are vital organs in eukaryotic cells, serving as both energy producers for important cellular processes and regulators of internal apoptotic pathways [6]. Therefore, targeting mitochondrial function may be an effective strategy for cancer treatment. The biological variance has been utilized as a foundation for the creation of mitochondria-targeting conjugates. Delocalized lipophilic cations (DLCs), including triphenylphosphonium (TPP) [7], dequalinium [8], and rhodamine 123 [9], have commonly been employed as molecules for targeting mitochondria. Several conjugates have been developed to specifically target mitochondria, with the majority being derived from TPP due to its highly efficient ability to target mitochondria [10]. For instance, TPP has been utilized to selectively transport well-known anticancer medications like doxorubicin [11] and cisplatin [12] to the mitochondria of tumor cells, as well as to improve the bioavailability and cytotoxic effects of promising antitumor drugs.

Ginseng is extensively utilized in numerous countries for the treatment and prevention of various diseases [13]. As the active metabolite of ginsenoside, (20*S*)-protopanoxadiol (PPD) is thought to play a crucial role in the pharmacological benefits of ginseng, such as neuroprotection, immune system enhancement, fatigue reduction, and, notably, antitumor effects [14]. Pharmacological experiments have confirmed that PPD inhibits the growth of multiple types of cancer cells. PPD has also exhibited obvious synergistic and attenuated effects on nude mice with human small-cell carcinoma tumors. In addition, PPD can inhibit the proliferation of cancer cells and induce their apoptosis in various types of cancer, including liver, stomach, leukemia, cervical, melanoma, nasopharyngeal, etc. [15]. As a result, by employing PPD as a precursor in the research and development of novel derivatives, it will be possible to both better understand the active mechanism of PPD and create novel medications for the treatment of different cancers. However, the efficacy of PPD in clinical applications is restricted by its poor water solubility and cell permeability. Based on the membrane permeability of TPP [16], this study hypothesized that the conjugation of TPP with PPD could further enhance its cell permeability and increase its potential to directly destroy cancer cells.

In this study, TPP was incorporated into the OH group at the C-3 site to increase the membrane permeability and anticancer efficacy of PPD. Transcriptomics and metabolomics were also employed to provide a systematic and detailed perspective on the induced antitumor mechanisms of CTPPPPD. Moreover, this study explored the antitumor effects of CTPPPPD in NSCLC and validated possible molecular mechanisms obtained from transcriptomics and metabolomics. The study findings provide a scientific rationale for the use of CTPPPPD in NSCLC therapy.

## 2. Results

### 2.1. Synthesis of CTPPPPD

**Figure 1:**
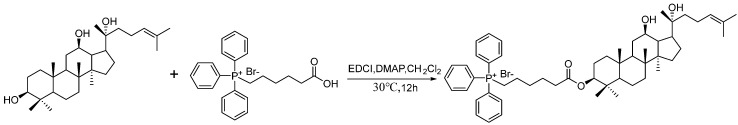
Synthesis pathway of CTPPPPD.

Figure 1 shows the synthesis pathway of CTPPPPD. The structural analysis of CTPPPPD was assessed using high-resolution mass spectrometry (HRMS) (Figure S1), proton nuclear magnetic resonance (^1^H NMR) (Figure S2), and Carbon-13 nuclear magnetic resonance (^13^C NMR) (Figure S3 and Figure S4). Compound 1: A white crystalline substance dissolved in ethyl acetate, with a yield of 78.3%. The nuclear magnetic resonance (NMR) spectrum was obtained using a 600 MHz instrument and pyridine as the solvent. The chemical shifts of the protons in the compound are as follows: 7.77 (m, 5H), 7.68 (m, 10H), 5.32 (1H, t, J = 6.5 Hz), 4.14(m, 2H), 3.92 (m, 1H), 1.65 (s, 3H), 1.63 (s, 3H), 1.44 (s, 3H), 0.97 (s, 3H), 0.93 (s, 3H), 0.88 (s, 3H), 0.87 (s, 3H), 0.81 (s, 3H).13C NMR (151 MHz, Pyr) *δ* 173.37(-COO-), 135.54(3C),134.58,134.51(6C), 131.04,130.96(6C), 131.1(-CH=CH-), 126.67(-CH=CH-), 123.50, 119.73, 119.16, 80.84, 73.34, 71.23, 56.40, 55.10, 52.02, 50.60, 48.90, 40.34, 38.93, 38.41, 37.49, 36.21, 35.34, 34.67, 32.38, 31.69, 30.64, 30.53, 28.48, 27.44, 27.17, 26.16, 25.08, 24.38, 23.35, 22.89, 18.77, 18.04, 17.39, 17.16, 16.65, 16.16. HRMS shows *m*/*z* values. The chemical formula is C54H76O4P+. The calculated value was 819.55, while the test value was 819.55111.

### 2.2. CTPPPPD Inhibiting A549 Cell Proliferation Activity

**Figure 2:**
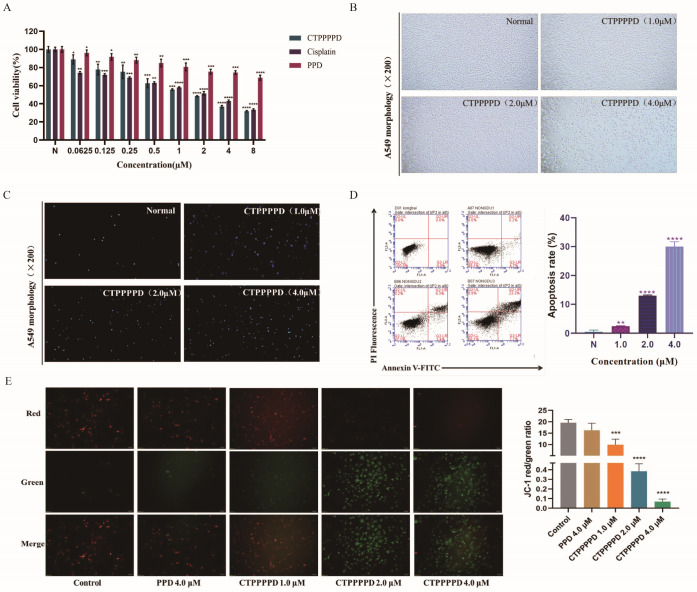
The effect of CTPPPPD on A549 cells. (**A**) Effects of CTPPPPD, PPD and Cisplatin on A549 cell proliferation. (**B**) Effects of CTPPPPD on A549 cell morphology observed under normal conditions. (**C**) Effects of CTPPPPD on A549 cell morphology observed by Hoechst 33,258 staining. (**D**) CTPPPPD induces A549 cell apoptosis. (**E**) Effects of CTPPPPD on A549 cell mitochondrial membrane potential detected by JC-1. *, **, *** and **** represent significant *p*-values ≤ 0.05, 0.01, 0.001, 0.0001, respectively.

The effect of CTPPPPD on the viability of A549 cells was assessed using the CCK8 assay. The viability of A549 cells was determined after they were exposed to varying concentrations of CTPPPPD for 24 h. Figure 2A illustrates that the CTPPPPD treatment caused significant anti-proliferative effects on A549 cells in a concentration-dependent manner. After 24 h, the A549 cells showed IC50 values of 54.56 ± 4.56 μmol/L, 1.65 ± 0.10 μmol/L, and 1.82 ± 0.25 μmol/L for PPD, CTPPPPD, and cis-platinum, respectively. These results suggest that the anti-proliferation effects of CTPPPPD on A549 cells are similar to that of cisplatin, and CTPPPPD is about 33-fold more effective than PPD.

The growth rate of A549 cells experienced a significant reduction after being treated with CTPPPPD for 24 h. Intracellular vacuoles became visible, along with a substantial amount of cell fragments floating on the surface of the culture medium. Moreover, as the concentration of CTPPPPD increased, there was a significant reduction in the number of A549 cells (Figure 2B). When cells undergo apoptosis, there is an increase in membrane permeability and a change in the structure of chromosomal DNA. This allows Hoechst 33,258 staining solution to bind more effectively to the DNA, resulting in higher blue fluorescence intensity in apoptotic cells compared to normal cells. The CTPPPPD group (1.0, 2.0, and 4.0 µM) exhibited a significant increase in nuclear dense staining spots of blue fluorescence, indicating that CTPPPPD may potentially induce apoptosis in A549 cells (Figure 2C).

Apoptosis is recognized as a primary mechanism of cancer cell death. FITC-Annexin V/propidium iodide (PI) assays and flow cytometric analysis were implemented to examine the potential of CTPPPPD to induce apoptosis. The findings indicated that CTPPPPD induced apoptosis in A549 cells (Figure 2D). After being treated with CTPPPPD (0, 1.0, 2.0, and 4.0 μM) for 24 h, the percentage of cells undergoing apoptosis was 0%, 4.9%, 12.7%, and 31.0%, respectively. The results showed that the CTPPPPD treatment increased the apoptotic rate of A549 cells in a concentration-dependent manner.

The ratio of red to green fluorescence intensity in JC-1 staining was decreased, indicating a decrease in mitochondrial transmembrane potential. Compared with the control group, PPD (4.0 µM) did not cause any change in mitochondrial membrane potential, while CTPPPPD (1.0, 2.0, 4.0 µM) significantly reduced the mitochondrial membrane potential of A549 cells in a dose-dependent manner. This indicated that CTPPPPD modified with PPD could further accelerate the apoptosis of A549 cells through mitochondria (Figure 2E).

### 2.3. Analysis of Gene Expression Patterns and Anti-A549 Cell Activity of CTPPPPD

#### 2.3.1. Quality Control of RNA-Seq Data

The transcripts of CTPPPPD and control samples were compared using RNA-seq analysis, with an emphasis on examining the accumulation of mRNA data obtained from the cDNA libraries. The transcriptome analysis of 12 samples produced 84.19 Gb of clean data in total; each sample contained over 6.09 gigabytes of clean data, with Q30 bases exceeding 92.44% (Table S1).

The clean reads obtained after quality control were then compared to the reference genomes to obtain reliable information for further evaluation. The rate of alignment between each sample and the reference genome ranged from 95.94% to 96.63% (Table S2).

#### 2.3.2. Differentially Expressed Genes (DEGs) in A549 Cells

**Figure 3:**
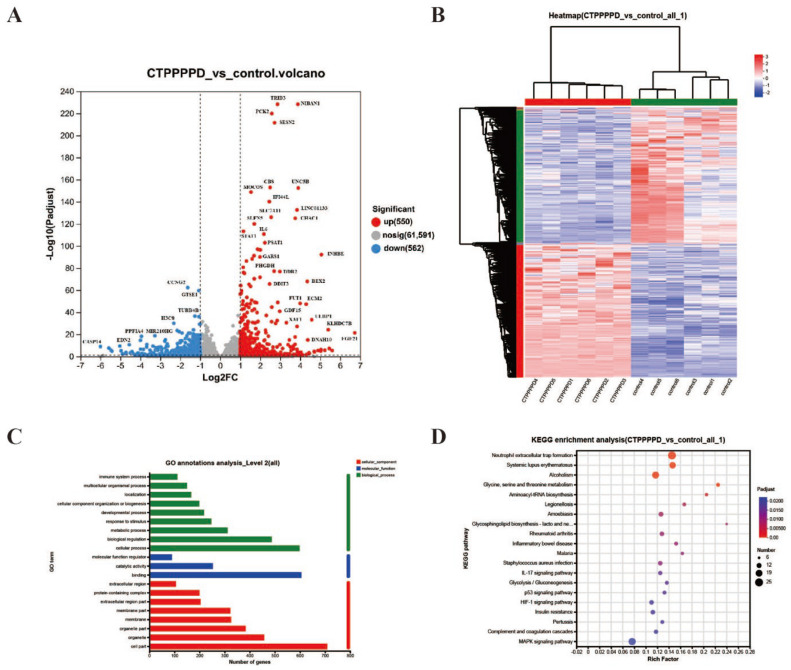
The screening results of DEGs from the model and CTPPPPD groups in A549 cell samples. (**A**) The volcanic plot of differential genes between the CTPPPPD and control groups. (**B**) Clustering heat map of differential genes between the CTPPPPD and control groups. (**C**) GO annotation analysis of CTPPPPD vs. control. (**D**) KEGG enrichment analysis of CTPPPPD vs. control.

To identify the DEGs in the treatment of CTPPPPD on A549, a threshold of *p*-adjust < 0.05 and up/down difference multiple > 2 was applied. This allowed us to screen for DEGs in all groups. Additionally, a volcano map was developed to visualize the DEGs. A total of 1112 DEGs were identified in the CTPPPPD group when compared to the control group (Figure 3A). Among these, 550 DEGs were up-regulated, while 562 were down-regulated. Then, the relative content changes of DEGs in each group were investigated in transcriptomics, and a heatmap of the relative content of DEGs of CTPPPPD for A549 treatment was drawn. The Euclidean approach and the complete linkage technique were employed for distance calculation and transcript analysis, respectively. The results showed that the mRNA expression profiles of the CTPPPPD and control groups were significantly clustered within the groups, suggesting that CTPPPPD treatment could significantly affect mRNA expression in A549 cells and play an anti-lung-cancer role (Figure 3B).

GO and KEGG pathway analyses were subsequently conducted to gain a better understanding of the function of the identified DEGs (Table S3). Figure 3C presents the results of the GO annotation analysis that compared the CTPPPPD and control groups. Significant enrichment was observed in the biological processes (BP) category for “cellular process”, “biological regulation”, and “metabolic process”. The classification of molecular function (MF) was significantly enhanced for “binding”, “catalytic activity”, and “molecular function regulator”. A significant increase was seen in the cell components (CC) category for the “cell part”, “organelle”, and “membrane part”. KEGG analysis showed significant enrichment in pathways, such as “neutrophil extracellular trap formation”, “MAPK signaling pathway”, “IL-17 signaling pathway”, “p53 signaling pathway”, and “Glycine, serine, and threonine metabolism” when comparing the CTPPPPD and control groups. Figure 3D presents 20 of the most important signaling pathways. The KEGG enrichment analysis revealed the significant enrichment of DEGs in various inflammatory and apoptosis pathways. These findings suggest that CTPPPPD can effectively prevent the growth of A549 tumor cells, which may be linked to the aforementioned pathways.

### 2.4. Analysis of Accumulated Metabolites of Anti-A549 Cell Activity of CTPPPPD

#### 2.4.1. Identification and Characterization of Metabolites

**Figure 4:**
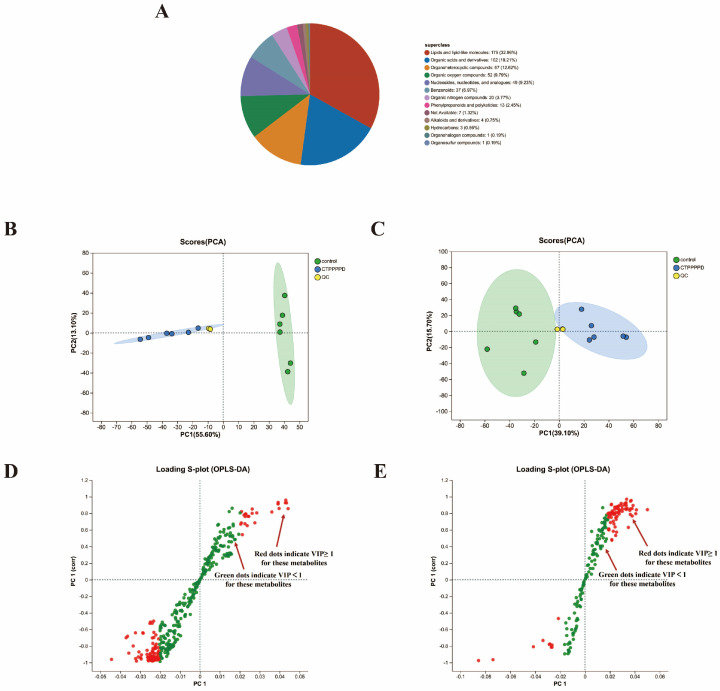
Overall analysis of DAMs. (**A**) Metabolite HMBD compound classification diagram. (**B**) PCA score plot of samples in cationic mode. (**C**) PCA score plot of samples in anionic mode. (**D**) OPLS-DA score plot of CTPPPPD vs. control in cationic mode. (**E**) OPLS-DA score plot of CTPPPPD vs. control in anionic mode.

The UHPLC-Q-TOF/MS analysis revealed 416 and 209 metabolites in the positive and negative ion modes, respectively (Table S4, Figure S5 and Figure S6). A database was used to annotate the obtained metabolites, and the results showed that 531 metabolite annotations were recorded in the human metabolome database (HMDB). Lipids and lipid-like molecules comprised 32.96% of the classifications, while organoheterocyclic compounds (12.62%), organic acids and derivatives (19.21%), and organic oxygen compounds (9.79%) accounted for the remaining classifications (Figure 4A).

#### 2.4.2. Differentially Accumulated Metabolites (DAMs) in A549 Cells

A multivariate statistical analysis was performed on the metabolomics data of A549 cells to compare the groups in the overall metabolic function changes. The PCA score plots showed significant differences between the groups in A549 cell metabolic profiles (Figure 4B,C). The OPLS-DA scoring plots revealed a significant difference between the CTPPPPD and control groups in both the positive and negative ion modes (Figure S7). These findings indicate that CTPPPPD treatment can effectively improve the metabolic disorder of A549 lung cancer cells. Likewise, the S-plots of OPLS-DA showed differences between the CTPPPPD and control groups in the metabolomics profiles of A549 cells, which made it possible to gather VIP values for the metabolites (Figure 4D,E).

**Figure 5:**
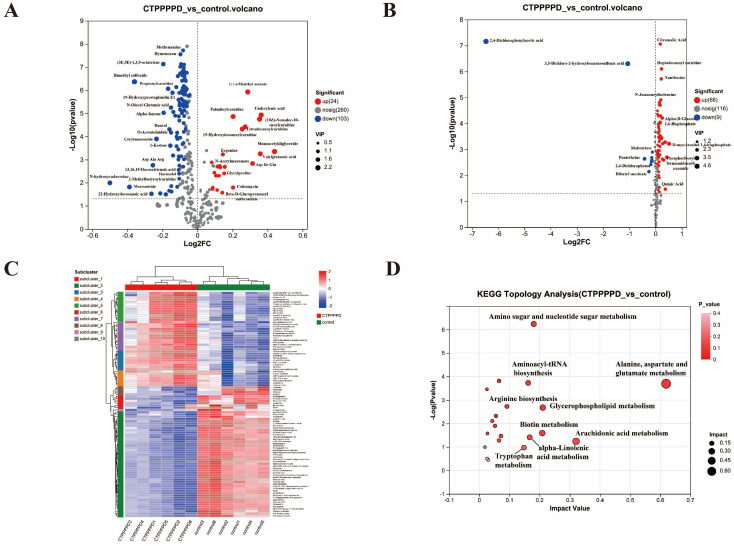
Overall analysis of DAMs. (**A**) The volcanic plot of DAMs between CTPPPPD and control groups in cationic mode. (**B**) The volcanic plot of DAMs between CTPPPPD and control groups in anionic mode. (**C**) Clustering heat map of the top 100 metabolites in CTPPPPD vs. control. (**D**) KEGG topology analysis of CTPPPPD vs. control.

A volcanic plot was developed to demonstrate the differences between the CTPPPPD and control groups in metabolites of cationic and anionic modes. The screening criteria included *p*-value < 0.05, vip-pred-OPLS-DA > 1, and up/down difference ratio > 1 (Figure 5A,B). The *x*-axis represents the log2FC, which is the fold change in metabolite expression between the two groups, while the *y*-axis represents the −log10 (*p*-value), which is the statistical test value for the difference in metabolite expression; accordingly, the greater the value, the more pronounced the difference in expression. Moreover, the left point represents the metabolite with a down-regulated expression difference, while the right point denotes the metabolite with an up-regulated expression difference. The greater the deviation to the left and right, as well as up and down, the more pronounced the difference in the expression level. The results showed that a total of 92 up-regulated metabolites and 112 down-regulated metabolites deserved further analysis in cationic and anionic modes (Table S5). The heat map in Figure 5C shows the results of the metabolite clustering analysis. Each column represents a sample, each row represents a metabolite, and the colors indicate the relative expression of metabolites within the sample group. The tree diagrams on the left and right represent the metabolite cluster and the names of the metabolites, respectively. The proximity of the branches in the metabolite cluster indicates the similarity in their expression levels. Moreover, the tree diagram for sample clustering is located at the top, while the sample names are listed at the bottom. The proximity of the sample branches reflects the similarity in the expression patterns of all metabolites in the two samples, i.e., the similarity in the trend of changes in metabolite expression levels. The results suggest that CTPPPPD can significantly affect the expression of metabolites in A549 cells. The KEGG topology analysis revealed that the different metabolites were associated with various metabolic pathways, such as “alanine, aspartate, and glutamate metabolism”, “arachidonic acid metabolism”, “glycerophospholipid metabolism”, “biotin metabolism”, “amino sugar and nucleotide sugar metabolism”, “alpha-Linolenic acid metabolism”, and “aminoacyl-tRNA biosynthesis” (Table S6). These metabolic pathways may play a crucial role in the anti-A549 cell activity of CTPPPPD (Figure 5D).

### 2.5. Integrated Analysis of CTPPPPD Effects on A549 Cells

#### 2.5.1. Analyzing Transcriptome and Metabolome Association in Metabolic Pathways

**Figure 6:**
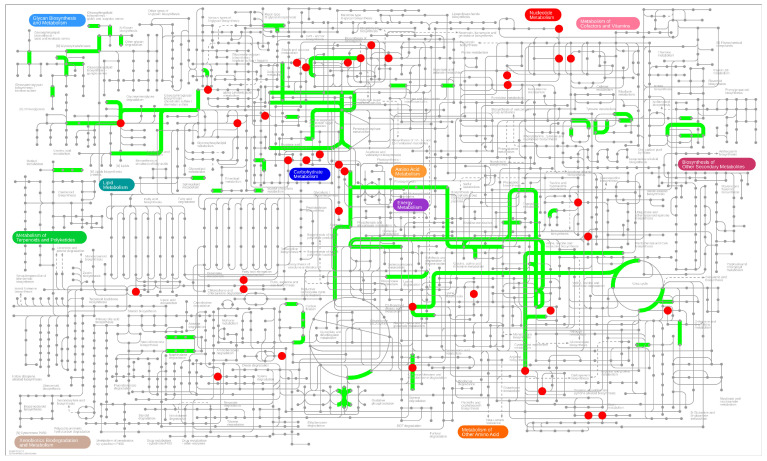
Integrated transcriptome and metabolome analysis in iPath 3.0. Red represents pathways annotated by the metabolic set and green represents pathways annotated by the gene set.

**Figure 7:**
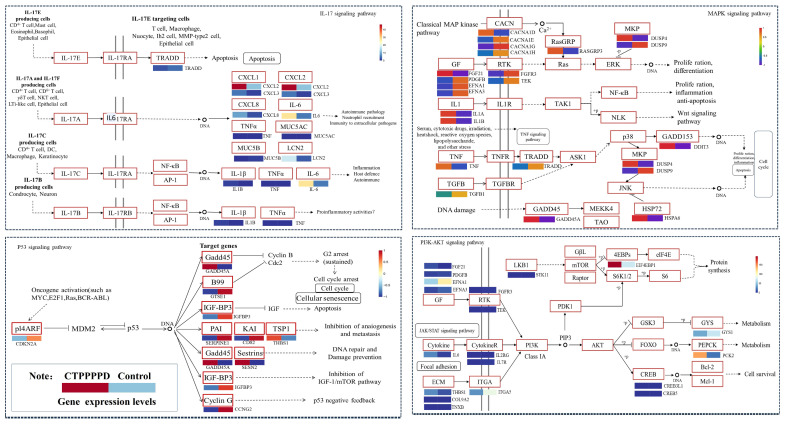
Effect of CTPPPPD on NSCLC response.

An extensive investigation (Figure 6) was conducted to further understand the protective effects of CTPPPPD on A549 cells by integrating DEGs and DAMs with iPath 3.0 (http://pathways.embl.de) (accessed on 21 May 2024). Compared to the control group, DEGs and DAMs showed significant enrichment in metabolic pathways, such as “carbohydrate metabolism”, “glycan biosynthesis and metabolism”, “energy metabolism”, and “amino acid metabolism” (Figure 7). The integrated transcriptome and metabolome analysis demonstrated that CTPPPPD contributes to the inhibition of NSCLC, primarily by influencing the synthesis and breakdown of sugar and amino acids in cancer cells.

#### 2.5.2. Effect of CTPPPPD on Important Relevant Pathways in NSCLC

As previously stated, many inflammation-associated pathways showed significant enrichment, including the IL-17 signaling pathway (mmu04657), TNF signaling pathway (mmu04668), MAPK signaling pathway (mmu04010) (Figure S8), PI3K-AKT signaling pathway (mmu04151) (Figure S9), and P53 signaling pathway (mmu04115) (Figure S10). Then, enrichment pathway maps and heatmaps were developed to examine how CTPPPPD affects NSCLC. When the experimental and control groups were compared, it was observed that the expression of multiple crucial genes was regulated within these inflammation-associated metabolic pathways. This indicates an inflammatory reaction in NSCLC after CTPPPPD induction (Figure 7). It is noteworthy that nine genes showed significant regulation in the P53 signaling pathway in the CTPPPPD group when compared to the control group. These genes included SESN2, IGFBP3, CDKN2A, GADD45A, GTSE1, THBS1, SERPINE1, CD82, CCNG2. After CTPPPPD treatment, 21 DEGs in the MAPK signaling pathway exhibited significant regulation. These genes included CACNA1E, FGF21, FGFR3, GADD45A, TGFB1, EFNA1, CACNA1G, DUSP9, PDGFB, CACNA1D, EFNA3, TRADD, TEK, DUSP4, DDIT3, HSPA6, RASGRP3, CACNA1H, and so on.

**Figure 8:**
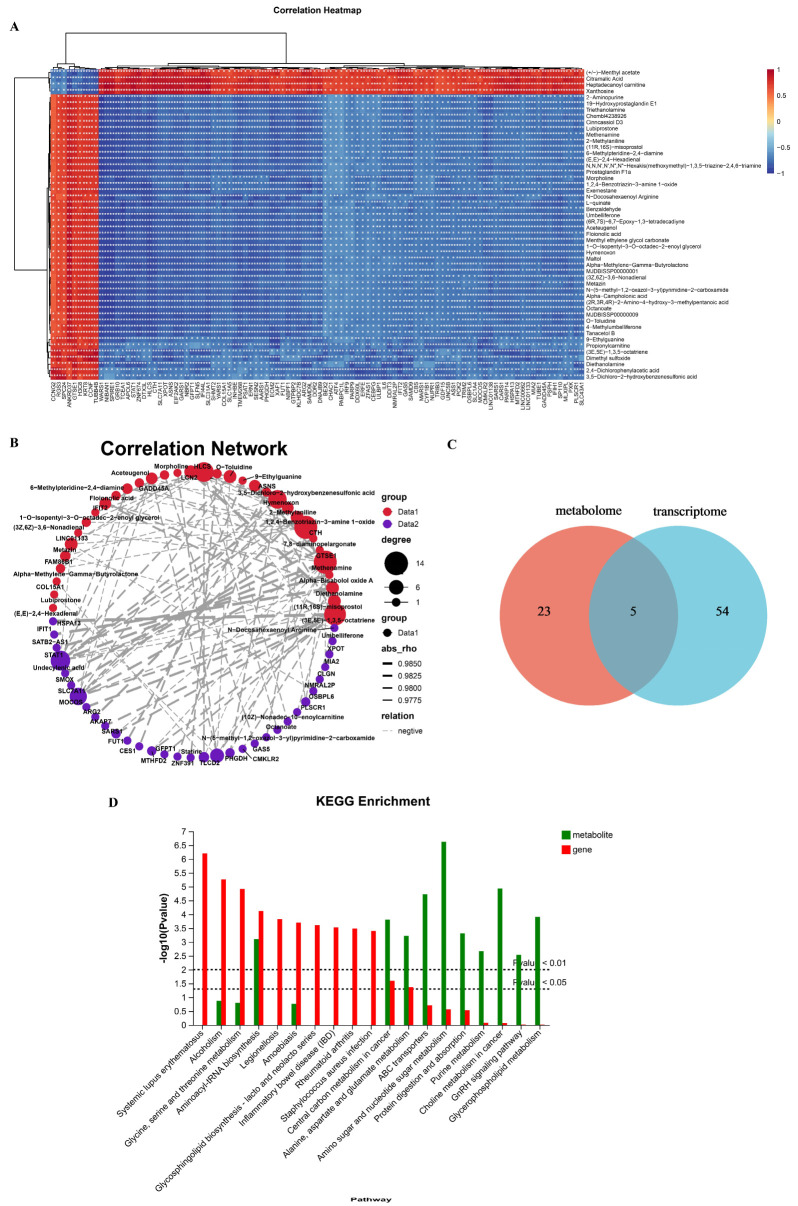
Integrated analysis of transcriptome and metabolome. (**A**) Correlation heatmap of the first 100 genes and top 50 metabolites between the CTPPPPD and control groups. (**B**) Correlation network of the top 100 pairs of genes and metabolites with significant differences between the CTPPPPD and control groups. (**C**) Venn diagram of KEGG pathway enrichment between the CTPPPPD and control groups. (**D**) Enrichment statistics for KEGG pathways of genes and metabolites in the CTPPPPD and control groups.

CTPPPPD intervention resulted in the significant regulation of 19 genes in PI3K-Akt signaling pathways, as compared to the control group (Figure 8 and Figure S9). These encompass various receptor genes, such as those belonging to the interleukin receptor family (IL7R, IL6, and IL2RG), the FGFs and FGFRs family protein (FGF21 and FGFR3), and genes that encode members of the translational repressor protein family EIF4EBP1. A significant difference was observed between the CTPPPPD and control groups in STK11, EFNA1, ITGA5, PDGFB, THBS1, CREB5, GYS1, EFNA3, CREB3L1, COL9A2, TEK, PCK2, and TNXB genes.

#### 2.5.3. Effects of CTPPPPD on Pathways Related to NSCLC Metabolism

**Figure 9:**
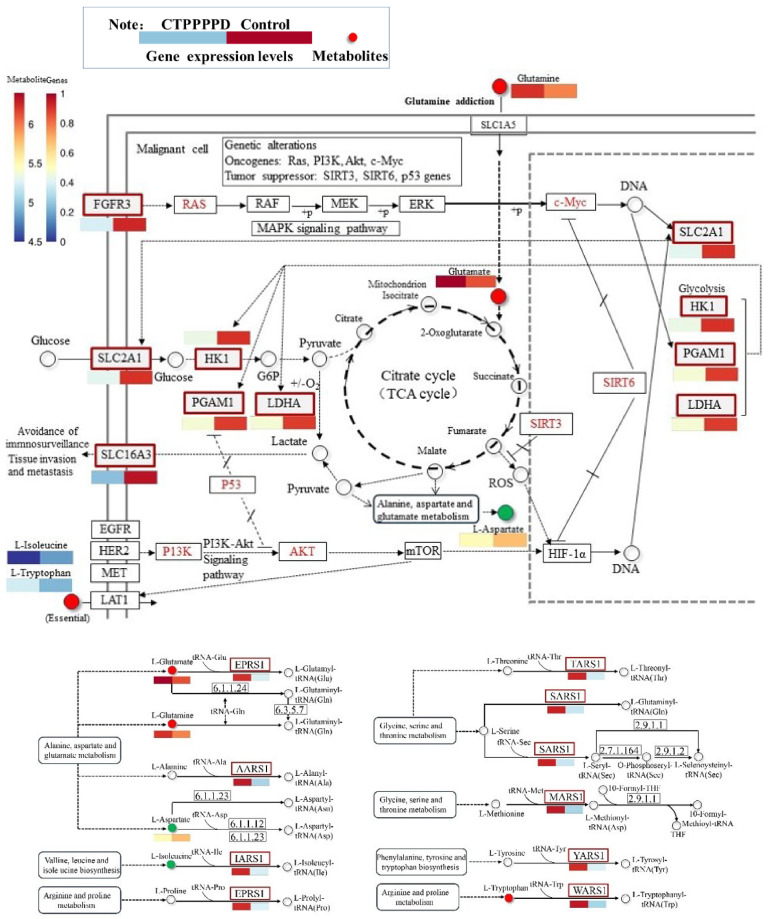
CTPPPPD against NSCLC in central carbon metabolism in cancer and aminoacyl-tRNA biosynthesis pathway.

To further investigate the effects of CTPPPPD on NSCLC, the significant differences in transcriptome expression levels of 100 genes and metabolic pathways involving 50 metabolites were analyzed. The Pearson algorithm was also employed to generate correlation heatmaps (Figure 8A). At the same time, correlation analysis was performed on all DEGs and DAMs; the Pearson algorithm was also used to obtain the correlation analysis data and draw the correlation network diagram (Figure 8B). In addition, the integrated analysis of metabolome and transcriptome yielded the Venn diagram and KEGG pathway enrichment statistics for metabolism and transcription (Figure 8C,D). Interestingly, there were five common pathways in the metabolome and transcriptome. The central carbon metabolism plays a crucial role in cancer development (Figure 9), aminoacyl tRNA biosynthesis is essential for protein synthesis, proximal tubule bicarbonate reclamation is important for maintaining acid–base balance, arginine biosynthesis is a key pathway for producing this amino acid, and alanine, aspartate, and glutamate metabolism are interconnected processes that play vital roles in cellular function. Table 1 presents the specific genes and metabolites involved in the pathway (Figures S11–S15). Importantly, after CTPPPPD treatment, SLC16A3 (ENSG00000141526), FGFR3 (ENSG00000068078), LDHA (ENSG00000134333), HK1 (ENSG00000156515), PGAM1 (ENSG00000171314), and SLC2A1 (ENSG00000117394) isogenes in the central carbon metabolism in cancer pathway and L-glutamine, L-tryptophan, L-glutamic acid, L-isoleucine, L-aspartic acid, and other metabolites significantly changed compared to the control group (Figure 8A). The results indicated that the intervention group, which received CTPPPPD, experienced changes in the metabolism of several amino acids (L-glutamine, L-tryptophan, L-glutamic acid, L-isoleucine, L-aspartic acid) in the aminoacyl-tRNA biosynthesis pathway, compared to the control group. Furthermore, the key factors in the biosynthesis pathway of aminoacyl tRNA, such as YARS1 (ENSG00000134684), MARS1 (ENSG00000166986), AARS1 (AARS1), SARS1 (ENSG00000031698), EPRS1 (ENSG00000136628), IARS1 (ENSG00000196305), WARS1 (ENSG00000140105), and TARS1 (ENSG00000113407), were affected.

#### 2.5.4. Quantitative Real-Time PCR (qRT-PCR)

**Figure 10:**
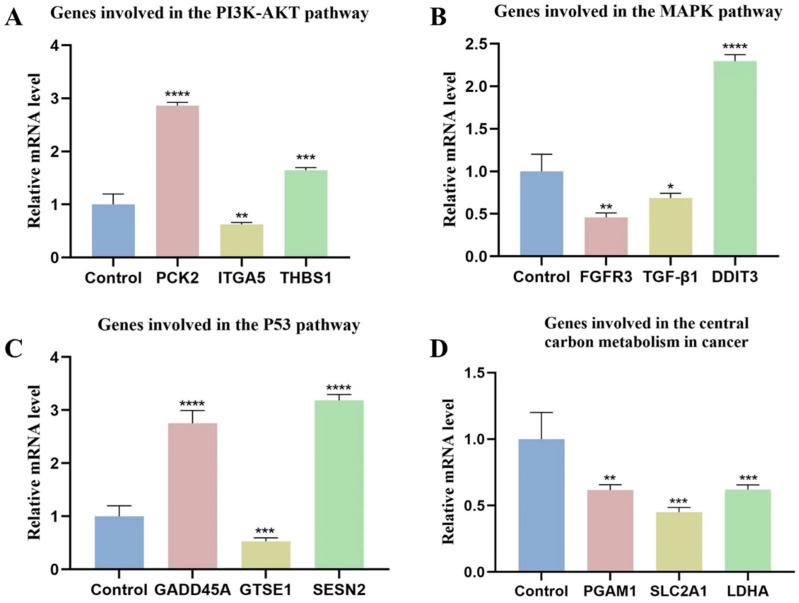
The qRT-PCR-based analysis was conducted to assess the expression levels of 12 selected unigenes. (**A**) Genes involved in the PI3K-AKT pathway. (**B**) Genes involved in the MAPK pathway. (**C**) Genes involved in the P53 pathway. (**D**) Genes involved in the central carbon metabolism in cancer. *, **, *** and **** represent significant *p*-values ≤ 0.05, 0.01, 0.001, 0.0001, respectively.

The qRT-PCR analysis was conducted to confirm the relative expression levels of 12 important genes related to PI3K-AKT, MAPK, and P53 response, as well as central carbon metabolism in cancer. The results were consistent with the transcriptome sequencing data. Notably, DDIT3, PCK2, THBS1, GADD45A, and SESN2 were found to be up-regulated in the control group but downregulated in the CTPPPPD group. In contrast, the expression level of FGFR3, TGF-β1, ITGA5, GTSE1, PGAM1, SLC2A1, and LDHA decreased in the control group but increased in the CTPPPPD group (Figure 10). These findings generally indicate that the anti-NSCLC effect of CTPPPPD therapy can be attributed to its influence on the expression of essential genes associated with pathways related to central carbon metabolism in cancer and aminoacyl-tRNA biosynthesis.

#### 2.5.5. Molecular Docking of CTPPPPD

**Figure 11:**
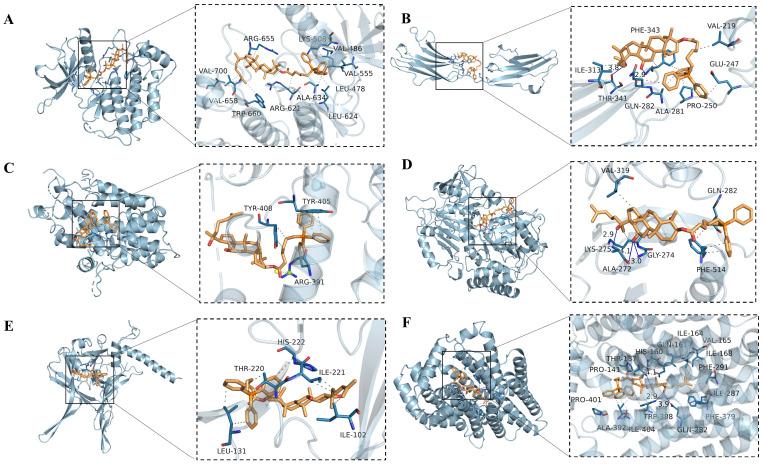
CTPPPPD molecular docking results for related proteins pertaining to central carbon metabolism in cancer. (**A**) Molecular docking diagram of FGFR3 (PDB code: 3GRW) vs. CTPPPPD. (**B**) Molecular docking diagram of FGFR3 (PDB code: 4K33) vs. CTPPPPD. (**C**) Molecular docking diagram of SESN2 (PDB code: 5CUF) vs. CTPPPPD. (**D**) Molecular docking diagram of PCK2 (PDB code: 5I67) vs. CTPPPPD. (**E**) Molecular docking diagram of TGF-β1 (PDB code: 5VQP) vs. CTPPPPD. (**F**) Molecular docking diagram of SLC2A1 protein (PDB code: 6THA) vs. CTPPPPD.

The molecular docking results showed that CTPPPPD could form hydrogen bonds with the FGFR3 receptor (PDB code: 4K33 and 3GRW), which is a key target involved in MAPK and central carbon metabolism in cancer pathways. The activity of protein ligands can effectively combine to pocket. Considering its combination for 7.9 kcal/mol, the binding energy is less than 7, and said compounds can be combined with protein pocket happen hydrophobic interaction. Then, through the analysis of 3D interaction, CTPPPPD formed hydrophobic interactions with amino acids VAL700, ARG655, LYS508, VAL486, VAL555, LEU478, LEU624, ALA634, ARG621, TRP660 and VAL658 of the protein. Their activity promotes the interaction between ligands and binds to protein pocket-forming complexes (Figure 11A). Similarly, the hydrophobic functional group of CTPPPPD formed hydrogen bond interaction with amino acids THR341, PHE343 of the protein, and CTPPPPD formed hydrophobic interaction with amino acids ILE313, VAL219, GLU247, PRO250, ALA281, GLN282 of FGFR3 protein (Figure 11B).

The hydrophobic functional group of CTPPPPD formed a salt bridge interaction with amino acid ARG391 and a hydrophobic interaction with amino acids TYR408 and TYR405 of the SESN2 (PDB code: 5CUF) in the P53 pathway (Figure 11C). The hydrophobic functional group of CTPPPPD formed a hydrogen bond interaction with amino acids GLY274, ALA272 and LYS275 of the protein, and CTPPPPD formed a hydrophobic interaction with amino acids VAL319, GLN282 and PHE514 of PCK2 protein (PDB code: 5I67) in the PI3K-AKT pathway (Figure 11D). CTPPPPD formed a hydrophobic interaction with amino acids LEU131, THR220, HIS222, ILE221, ILE102 of TGF-β1 protein (PDB code: 5VQP) in the MAPK pathway (Figure 11E). The hydrophobic functional group of CTPPPPD formed a hydrogen bond interaction with amino acids THR37, TRP388, GLN282 of the protein, and CTPPPPD formed a hydrophobic interaction with amino acids PRO401, PRO141, HIS160, GLN161, ILE164, VAL165, ILE168, PHE291, ILE287, PHE379, ILE404, ALA392 of SLC2A1 protein (PDB code: 6THA) in central carbon metabolism in cancer (Figure 11F). The binding energy values of CTPPPPD and the core targets are shown in Table 2.

## 3. Discussion

Lung cancer ranks as the second most prevalent form of cancer globally and remains the leading cause of cancer-related deaths, posing a considerable burden on global public health [17]. NSCLC makes up over 80% of all cases of lung cancer globally, with small-cell lung cancer (SCLC) being the other main type. Advances in surgery, radiotherapy, chemotherapy, molecular targeted therapy, immunotherapy, and other technologies over the past 20 years have significantly prolonged the lives of many cancer patients and greatly improved their quality of life [18]. Nevertheless, owing to its progression and challenging curability, the mean 5-year survival rate among patients with lung cancer is only 19% [19]. Therefore, it is necessary to develop novel, low-toxicity, high-efficiency drugs that can combat lung cancer drug resistance.

Ginseng is a traditional Chinese herb, known as the king of herbs [20]. Protopanoxadiol (PPD) is an important active component of ginseng, which plays an important role in the proliferation, apoptosis, differentiation, metastasis, and angiogenesis of tumor cells [21]. As stated earlier, TPP exhibits a robust ability to specifically target mitochondria and enhance the ability of potential antitumor drugs to penetrate the cell membrane and exert cytotoxic effects. Therefore, this study employed the CCK8 assay, Hoechst33258, and Annexin V-FITC/PI apoptosis assay to successfully synthesize a novel structure of CTPPPPD and confirm its potent anti-NSCLC activity to further improve the bioavailability of PPD. The results indicated that the anti-A549 activity of CTPPPPD (IC50 = 1.65 ± 0.10 μmol/L) was about 33-fold higher than that of PPD (IC = 54.56 ± 4.56 μmol/L).

Metabolomics and transcriptomics analyses were conducted to further investigate the mechanism of action of CTPPPPD against NSCLC. Proteomics, an emerging discipline that has developed rapidly alongside post-genomic metabonomics analysis, is a crucial component of systems biology [22]. Metabolomics is commonly employed for the qualitative and quantitative analysis of small-molecule metabolites (with a relative molecular mass of less than 1000) in organisms or cells. In this study, UPLC-MS/MS metabolomics was used to comprehensively and systematically identify and analyze the metabolites of A549 cells treated with CTPPPPD. The additional KEGG analysis findings demonstrated a significant correlation between the metabolites and several metabolic pathways, including “alanine and aspartate and glutamate metabolism”, “arachidonic acid metabolism”, “glycerophospholipid metabolism”, “biotin metabolism”, “amino sugar and nucleotide sugar metabolism”, “alpha-Linolenic acid metabolism”, and “aminoacyl-tRNA biosynthesis”. These correlations suggest that these pathways could potentially serve as the mechanisms through which CTPPPPD treats NSCLC.

Transcriptomics typically refers to the entirety of RNA molecules that can be transcribed within a cell at a specific state, encompassing both messenger RNA (mRNA) and non-coding RNA. Studies have shown that the new gene, gene expression, and quantitative analysis are commonly used to identify the transcription of this experimental method [23]. In this study, a total of 1112 DEGs were screened in the CTPPPPD group, 550 and 562 of which were up-regulated and down-regulated, respectively. Interestingly, the mRNA expression profiles in the CTPPPPD and control groups were significantly clustered within the group, indicating that CTPPPPD treatment could significantly affect the mRNA expression of A549 cells and exert an anti-lung-cancer effect. The KEGG analysis was performed to compare DEGs between the CTPPPPD and control groups, and the results showed that “PI3K-AKT”, “MAPK signaling pathway”, “p53 signaling pathway”, and other pathways were significantly enriched. These results indicated that CTPPPPD could effectively inhibit the proliferation of A549 tumor cells, which may be closely related to the above pathways.

The PI3K/Akt signaling pathway is a core signal transduction system in cellular physiological and pathological processes. It interacts with a variety of other signaling pathways to jointly regulate cell survival, proliferation, migration, metabolism, and other functions [24]. Studies have shown that the activation of this pathway can promote the proliferation and survival of lung cancer cells, inhibiting cell apoptosis, which helps tumors grow and spread [25]. The high expression of PCK2 in NSCLC may serve as a positive prognostic indicator [26]. This study demonstrated a significant increase in PCK2 after the CTTPPPD treatment, suggesting that it may be influenced by the promotion of tumor cell metabolism and the cell-cycle process, leading to a positive therapeutic effect on NSCLC. Other studies have shown that ITGA5 is an early prognostic factor for NSCLC [27], and the high expression of ITGA5 indicates poor prognosis and a higher risk of tumor metastasis [28]. ITGA5 was significantly down-regulated after CTTPPPD administration, indicating that CTTPPPD administration can effectively inhibit lung cancer metastasis. THBS1 plays a crucial role in suppressing tumor growth, cell migration, and angiogenesis. In NSCLC patients, lower expression of THBS1 indicates a worse prognosis. The administration of CTTPPPD may increase THBS1 expression, leading to the inhibition of tumor growth and metastasis, thereby improving treatment outcomes [29].

The PI3K/Akt and MAPK/ERK pathways are co-activated under the influence of multiple growth factors and extracellular signals, such as through EGFR (epidermal growth factor receptor). Akt can indirectly influence the activation of ERK, and there is also reciprocal inhibition, where ERK can inhibit certain components of the PI3K/Akt pathway. This demonstrates the dynamic equilibrium and mutual regulation of these two pathways within the intracellular signaling network. The MAPK signaling pathway also plays an important role in the development of cancer. Studies have shown that inhibiting the activation of the MAPK pathway in mice can effectively hinder the progression of NSCLC, thus serving as a potential treatment for cancer [30]. It has been reported that the overexpression of FGFR3 may promote the invasion and metastasis of lung cancer cells by activating the MAPK pathway [31]. After CTTPPPD treatment, a significant reduction in FGFR3 gene expression was observed, which may help to reduce the risk of lung cancer metastasis. TGF-β1 is highly expressed in NSCLC patients, which can promote the growth, invasion, and metastasis of NSCLC [32]. The CTTPPPD administration significantly reduces the expression of TGF-β1, indicating that CTTPPPD can reduce the migration and local invasion of lung cancer cells. The study revealed that the reduced expression of DDIT3 in lung cancer shortens the survival time of patients [33]. This study showed that CTTPPPD can significantly raise the expression of DDIT3, which may inhibit the growth of lung cancer cells and diffusion.

The PI3K/Akt signaling pathway uses a variety of mechanisms to affect p53, one of the major cancer suppressors involved in the regulation of cell-cycle arrest, apoptosis, and genomic stability [34]. Closely related to NSCLC, p53 is one of the most commonly mutated genes in this type of cancer [35]. For example, Akt can directly phosphorylate MDM2 to promote MDM2-mediated p53 degradation. In addition, Akt activation can also inhibit p53 stability and activity in other ways, affecting the cell cycle and apoptosis. GADD45A is a downstream gene regulated by p53, and its abnormal expression is closely related to lung, breast, pancreatic, and prostate cancers. The increased expression of GADD45A can significantly promote the apoptosis of A549 cells [36]. The up-regulation of GADD45A after CTTPPPD administration may cause remarkable antitumor effects on lung A549 cells by enhancing the apoptotic pathway. GTSE1 reduces the p53-mediated tumor suppressor function by inhibiting the expression of p53. Therefore, it plays a role in the proliferation and survival of lung cancer cells. The up-regulation of GTSE1 in lung cancer patients is associated with disease progression and a reduced survival rate [37]. It may inhibit the proliferation of lung cancer cells and exhibit anti-NSCLC effects. The up-regulation of SESN2 may inhibit the growth of lung cancer cells and slow down the progression of tumors by affecting the cell cycle and cell proliferation [38]. This study showed that the CTTPPPD administration significantly increased the expression of SESN2. This suggests that CTTPPPD may induce cell-cycle arrest by affecting cell-cycle progression in lung cancer cells and, thereby, inhibit the proliferation of tumor cells.

The TCA cycle, also known as the Krebs cycle, is a key metabolic pathway that provides energy for cellular metabolism [39]. The TCA cycle engages in the metabolism of glucose and also coordinates the metabolism of glutamine and other amino acids and fatty acids to ensure the effective use of intracellular metabolites [40]. LDHA is a crucial enzyme in the process of glycolysis, and increasing its activity may lead to higher levels of lactate production. This, in turn, can facilitate the growth and proliferation of tumor cells [41]. Studies have shown that LDHA overexpression is associated with a lower survival rate in NSCLC patients [42]. This study showed that CTTPPPD could significantly reduce LDHA expression, suggesting that CTTPPPD may inhibit the glycolytic pathway of tumor cells and reduce energy production and, thereby, inhibit the growth of tumor cells. PGAM1 is an oncogene that activates the TGF-β signaling pathway in NSCLC to increase the proliferation and invasion of cancer cells [43]. CTTPPPD may inhibit the activation of the TGF-β signaling pathway and reduce the proliferation and invasion of cancer cells by reducing the expression of PGAM1. SLC2A1 is a transporter that mainly mediates the regulation of energy metabolism in tumors. While providing energy for tumors, SLC2A1 promotes tumor growth and metastasis [44]. Studies have reported a negative correlation between the up-regulation of SLC2A1 and the overall survival of lung cancer patients [45]. On the other hand, the downregulation of SLC2A1 expression after CTTPPPD administration may inhibit tumor growth and metastasis by reducing glucose uptake and energy supply of tumor cells. Studies have shown the expression of FGF ligands and receptors plays a major role in the development of lung cancer. Specifically, fibroblast growth factor receptor 3 (FGFR3) is expressed primarily in epithelial cells and, to a lesser extent, in interstitial cells. However, a study reported the significant up-regulation of FGFR3 in bone metastasis of lung adenocarcinoma [46]. In this study, the expression of FGFR3 was significantly reduced following CTPPPPD administration. Other studies have shown that SLC16A3 is highly expressed in lung adenocarcinoma tissues and is associated with poor prognosis in patients [47]. Another study discovered a significant decrease in the expression of SLC16A3 following CTPPPPD administration, indicating that CTPPPPD is involved in cancer and is closely associated with the central carbon metabolism pathway in cancer.

In this study, the administration of a certain dose of CTPPPPD significantly reduced the expression of SLC16A3. This study demonstrated the significance of metabonomics and transcriptome analysis in understanding the central carbon metabolism in cancer. It also highlighted the importance of pathways, such as Aminoacyl-tRNA biosynthesis, proximal tubule bicarbonate reclamation, arginine biosynthesis, alanine, aspartate, and glutamate metabolism, in the anti-NSCLC mechanism of CTTPPPD.

## 4. Materials and Methods

### 4.1. Chemicals and Reagents

(20*S*)-protopanaxadiol was derived from ginsenosides as a starting material via saponin grouping (5%NaOH) and high-temperature alkali degradation (170 °C,10% NaOH, 36 h) in the lab, with a purity exceeding 95%, as confirmed by HPLC analysis. Chemical reagents were provided from Macklin Biochemical Technology (Shanghai, China) and utilized in their original form. The reactions were monitored through TLC analysis using pre-coated silica gel HSGF254 plates, and a 10% sulfuric acid solution was employed as a visualizing reagent. Silica gel (200–300 mesh, Qingdao, China) was utilized for column chromatography. The melting point was determined using a YRT-3 melting point meter (Tianjin, China). The ^1^H and ^13^C NMR spectral data were collected with a Bruker Avance 500-MHz spectrometer from Bruker Corporation (Karlsruhe, Baden-Württemberg, Germany), using TMS in pyridine d5 as an internal standard. High-resolution mass spectrometry detection was performed on a Triple TOF 5600+ system coupled with an electrospray ionization (ESI) source from AB SCIEX Corporation (Boston, MA, USA).

### 4.2. Preparation of CTPPPPD

The compound (5-Carboxypentyl) triphenylphosphonium bromide (CTPP) (194 mg, 0.36 mmol) was dissolved in 5 mL of anhydrous dichloromethane and added to 0.30 mmol of (20*S*)-propanaxanediol (PPD) dichloromethane solution (10 mL). The reaction occurred at 30 °C, with the progress of the reaction being observed using a thin-layer plate. Following the reaction, the solvent was concentrated in vacuo, and the desired CTPPPPD compound was separated using column chromatography (DCM/methanol 30:1).

### 4.3. Cell Culture

The A549 human lung epithelial cells were provided from the Chinese Academy of Medical Sciences (CAMS) and were cultured in Roswell Park Memorial Institute (RPMI) 1640 (Gibco, Invitrogen, Carlsbad, CA, USA) with the addition of 10% fetal bovine serum (HyClone Laboratories, Logan, UT, USA), 100 U/mL of penicillin, and 100 U/mL of streptomycin (Gibco, Invitrogen, Carlsbad, CA, USA) at 37 °C in a humidified atmosphere of 5% CO_2_. The A549 cells were cultivated until they reached an approximate confluence of 80%, sustained with fresh medium, as previously mentioned, and sub-cultured every 2 to 3 days.

A CCK8 cytotoxicity assay was employed to measure cell viability, according to the previously described method [48]. A549 cells were seeded in 96-well plates at a density of 1 × 10^4^ cells/well and cultured overnight. Subsequently, the cells were exposed to CTPPPPD at varying concentrations (0.0625 μmol/L, 0.125 μmol/L, 0.25 μmol/L, 0.5 μmol/L, 1.0 μmol/L, 2.0 μmol/L, 4.0 μmol/L, and 8.0 μmol/L) for 24 h. After the treatment, 10 μL of CCK8 was added to each well and incubated at 37 °C for more than 1–2 h. The absorbance was then measured at 450 nm using an infinite E plex microplate reader from tecan Corporation (Männedorf, Zürich, Switzerland). The cell viability was calculated as the percentage of CCK8 reduction relative to the absorbance of the control cells. All experiments were conducted with three replicates.

### 4.4. Effect of CTPPPPD on A549 Cell Morphology

A549 cells at a density of 5 × 10^5^ were plated in 6-well plates and incubated for 24 h. Subsequently, the cells were treated with 1.0 μmol/L, 2.0 μmol/L, and 4.0 μmol/L of the CTPPPPD solution for another 24 h. Following the microscopic observation of cell morphology, the previous remedy was eliminated, and 1 mL of Hoechst 33,258 staining solution was added to every well. The cells were then placed in a 5% CO_2_ saturated humidity incubator at 37 °C for 20 to 30 min. Subsequently, the staining solution was removed, and the cells were washed 2–3 times with PBS buffer for 3–5 min before fluorescence detection under an inverted fluorescence microscope.

### 4.5. Apoptosis Analysis

Flow cytometry was employed to measure apoptotic cells after they were stained with FITC-Annexin V/PI, according to the method described in a previous study [49]. In brief, A549 cells were treated with 1.0 μmol/L, 2.0 μmol/L, and 4.0 μmol/L of CTPPPPD for 24 h in 6-well plates. The adherent and suspended cells were collected, washed twice with precooled PBS buffer, and re-suspended in 1 mL of binding buffer (1×). Then, 100 µL of the cell suspension was mixed with 5 µL of V-FITC and incubated at room temperature in the dark for 5 min. Following this, 5 µL of PPI and 400 µL of PBS were added, and the samples were immediately analyzed using flow cytometry. The obtained data were analyzed in BD Accuri C6.

### 4.6. Mitochondrial Membrane Potential (JC-1) Detection

According to the operation method of mitochondrial membrane potential (JC-1) detection kit, JC-1 working solution was added to 6-well plates treated with different concentrations of CTPPPPD (1.0, 2.0, 4.0 μM) and PPD (4.0 μM) and incubated at 37 °C for 30 min. At the end of incubation, the cells were washed twice with JC-1 buffer (1×) and observed under a fluorescence microscope.

### 4.7. Extraction of Total RNA and Transcriptome Analysis

RNA was isolated from the A549 cells utilizing TRIzol^®^ Reagent (Invitrogen, Carlsbad, CA, USA) in accordance with the manufacturer’s instructions. Transcriptome analysis was performed on six biological replicates for the CTPPPPD-treated or untreated groups using RNA sequencing (RNA-seq). Then, the 5300 biological analyzer from Agilent Corporation (Palo Alto, California, USA) was used to assess the integrity of RNA, and the Nanodrop-2000 spectrophotometer from Nanodrop Technologies (Wilmington, DE, USA) was used for quantitative analysis. Mayo Bio-Pharm (Shanghai, China) was asked to prepare and sequence RNA-seq libraries by using the Illumina Novaseq 6000 (San Diego, CA, USA) platform. The reference genome was obtained from http://asia.ensembl.org/Mus_musculus/Info/Index (accessed on 17 September 2023).

The gene expression level in each sample was measured using the transcript per million reads (TPM) method [50]. The DEGs were identified based on the *p*-adjusted (padj) < 0.05 and |log2 fold change| > 1 standard [51]. The DEGs were analyzed through the Majorbio cloud platform tool available at https://cloud.majorbio.com/ (accessed on 17 September 2023). This website provides complimentary online entry to the GO and KEGG databases.

### 4.8. Untargeted LC-MS Metabolomics Analysis

A total of 1 × 10^6^ cells per ml were cultured in culture flasks overnight to make the cells adhere to the wall. The cells were categorized into two groups: the normal group and the CTPPPPD group. Cells in the CTPPPPD group were treated with 2.0 μmol/L of CTPPPPD. Each group consisted of 6 parallel samples. After 24 h of CTPPPPD administration, the cells were washed three times with precooled PBS, and 1 mL of cold methanol–water (4:1) solution at −80 °C was added to the cell samples to quench the enzymatic reaction. The cells were transferred to a precooled 1.5 mL centrifuge tube with a cell scraper, and the cells were broken using a cell breaker (operated on ice). After centrifugation for 10 min (12,000 r·min^−1^, 4 °C), the supernatant was collected, transferred to a new 1.5 mL centrifuge tube, and dried using a nitrogen blower. The samples were freeze-dried, with a 200 L of methanol solution added and dissolved. After vortexing and centrifuging at 12,000 rmp for 15 min at 4 °C, the supernatant (60 L) was automatically transferred to the glass sampler in the small bottle. At the same time, 15 µL of the solution was mixed from each of the 12 samples to prepare QC samples.

Untargeted metabolomics analysis was performed as previously established [52]. Briefly, the ultra-high-performance liquid chromatography–quadrupole time-of-flight mass spectrometry (UHPLC-Q-TOF/MS) system was employed to analyze the studied metabolites. A Vanquish Horizon System (Thermo Scientific, Waltham, MA, USA) coupled with an AB SCIEX Triple TOF 5600 System (AB SCIEX, Framingham, MA, USA) was used to analyze the metabolic profiling in both ESI-positive and ESI-negative ion modes. The sample was fractionated using a BEH C18 column (1.7 μm, 2.1 × 100 mm) with 10 microliters and then subjected to mass spectrometry analysis. The mobile phase-A consists of water with 0.1% formic acid, while the mobile phase-B is a mixture of acetonitrile and isopropanol in a 1:1 ratio, also containing 0.1% formic acid. A range of *m*/*z* 50–1000 was scanned to acquire sample quality spectrum signals using positive and negative ions in scanning mode. The cations were sprayed with a voltage of 5000 V, while the anions were sprayed with a voltage of 4000 V. Additionally, a bunch of anions were sprayed with a voltage of 80 V. The spray gas pressure was set at 50 psi, with auxiliary heat at 50 psi, gas curtain at 30 psi, and gas ion source heating temperature at 500 °C. The loop collision energy ranged from 20 to 60 V.

The initial LC-MS data were brought into Progenesis QI (Waters Corporation, Milford, CT, USA) for baseline filtering, peak identification, integration, retention time correction, and peak alignment, and, ultimately, a normalized data matrix was acquired. Simultaneously, MS mass spectrum data were compared to the public metabolic databases HMDB (http://www.hmdb.ca/) (accessed on 23 September 2023). and Metlin (https://metlin.scripps.edu/) (accessed on 23 September 2023). to retrieve information on metabolites. The pre-processed data were uploaded to the Megbio cloud platform (https://cloud.majorbio.com) (accessed on 23 September 2023). for data analysis. Metabolites with VIP > 1 and *p* < 0.05 were chosen as significant based on t-tests conducted using Metaboanalyst 4.0 (http://www.metaboanalyst.ca/) (accessed on 23 September 2023). Metabolic pathways were established using KEGG (http://www.genome.jp/kegg/pathway.html) (accessed on 23 September 2023). Metabolic pathways with a *p*-value of less than 0.05 were considered to be significantly influenced by CTPPPPD.

### 4.9. Real-Time Quantitative PCR (RT-qPCR)

The isolation of total RNA samples was carried out in accordance with the guidelines provided in Section 4.6. Before cDNA synthesis, the purity and concentration of RNA were evaluated using the μDrop Plate (Thermo Scientific, Juensuu, Finland). Afterward, reverse transcription and PCR were performed with the HiScript II Reverse Transcription Kit (Vazyme, Nanjing, China). The expression levels were assessed utilizing SYBR Green with an ABI QuantStudio 3 Real-Time PCR Instrument (Thermo Fisher, Waltham, MA, USA). The analysis was performed three times for each sample. The relative quantification analysis was standardized to GAPDH and computed utilizing the 2^−ΔΔCt^ technique. The primer sequences used in this study can be found in Table S7 of the Supplementary Materials.

### 4.10. Molecular Docking

The Autodock-vina 1.1.2 software was employed to investigate the binding of CTPPPPD to the PI3K-AKT, MAPK, P53, and central carbon metabolism pathways in cancer-related receptors. The 3D structures of PCK2 targets in the PI3K-AKT pathway (PDB code: 5I67), FGFR3 and TGF-β1 targets in the MAPK (PDB code: 4k33 and 5VQP), SESN2 targets in the P53 (PDB code: 5cuf), the SLC2A1 target of central carbon metabolism in cancer (PDB code: 6THA), and CTPPPPD were imported, and the interaction of the butt pose was analyzed in the pymol 2.3.0 software package.

### 4.11. Statistical Analysis

Data were presented as the mean ± standard deviation of six independent replicates. Statistical analysis was conducted in GraphPad Prism 9.5.0 from GraphPad Software Inc., (San Diego, California, USA) to identify significant variances in comparison to the corresponding controls within each experimental group. A significance threshold of *p* < 0.05 was employed to denote statistical significance.

## 5. Conclusions

The study findings suggested that the main mechanism of action of the novel CTPPPPD compound against NSCLC may be the interaction of the PI3K-AKT, MAPK, and p53 signaling pathways. CTPPPD has the potential to enhance the metabolic processes of cancer cells and affect the progression of the cell cycle, resulting in heightened cell membrane permeability, disruption of intracellular osmotic balance, impairment of the electron transport chain, disruption of cellular energy metabolism, reduced tumor cell viability, and reduced cell death. CBPE can significantly affect the metabolism of various substances, including alanine, glutamic acid, aspartic acid, arachidonic acid, glycerol phospholipid, amino sugar, nucleotide of sugar, ammonia acyl tRNA biosynthesis, and other metabolic pathways, within the cell. This allows CTPPPPD to inhibit NSCLC growth through multiple mechanisms. CTPPPD is a major potential antitumor agent for NSCLC. However, further studies are needed to explore the potential of CTPPPPD in treating NSCLC in vivo, as well as its potential applications in the food industry.

## Figures and Tables

**Table 1:** Mechanisms through which CTPPPPD disrupts the simultaneous enrichment of metabolome and transcriptome in A549 cells.

Pathway	Metabolites	Gene Symbol
Central carbon metabolism in cancer	L-Glutamine; L-Tryptophan; L-Glutamic Acid; L-Isoleucine; L-Aspartic Acid	SLC16A3; FGFR3; LDHA; HK1; PGAM1; SLC2A1
Aminoacyl-tRNA biosynthesis	L-Glutamine; L-Tryptophan; L-Glutamic Acid; L-Isoleucine; L-Aspartic Acid	YARS1; MARS1; AARS1; SARS1; EPRS1; IARS1; WARS1; TARS1
Proximal tubule bicarbonate reclamation	L-Glutamine; L-Glutamic Acid	AQP1; ATP1A3; PCK2
Arginine biosynthesis	L-Aspartic Acid; L-Glutamine; L-Glutamic Acid	NAGS; ASS1; ARG2
Alanine, aspartate and glutamate metabolism	N-Acetylaspartylglutamic Acid; L-Glutamine; L-Glutamic Acid; L-Aspartic Acid	ASNS; GFPT1; ASS1; RIMKLA

**Table 2:** The binding energy values of CTPPPPD and the core targets.

Gene	PDB ID	Compound	Binding Affinity (kcal/mol)
FGFR3	4K33	CTPPPPD	−7.9
FGFR3	3GRW	CTPPPPD	−7.1
SESN2	5CUF	CTPPPPD	−7.3
PCK2	5I67	CTPPPPD	−9.0
TGF-β1	5VQP	CTPPPPD	−6.9
SLC2A1	6THA	CTPPPPD	−9.8

## Data Availability

The original contribution presented in this study is included in the article/Supplementary Materials, and the raw RNA-seq data are freely available in the NCBI database under accession no. PRJNA1142464.

## Supplementary Materials

The following supporting information can be downloaded at: https://www.mdpi.com/article/10.3390/molecules29174275/s1, Table S1: Sequencing data statistics; Table S2: Results were compared with reference genomes; Table S3: GO results of all the enriched terms of DEGs from the comparison of CTPPPPD versus Control; Table S4: Total ion count and identification statistics; Table S5: DAMs from the comparison of CTPPPPD versus Control; Table S6: Enrichment of KEGG metabolic pathway of DAMs from the comparison of CTPPPPD versus Control; Table S7: Primer information of genes used for qPCR validation; Figure S1: HRMS of CTPPPPD; Figure S2: ^1^H NMR of CTPPPPD; Figure S3: ^13^C NMR of CTPPPPD; Figure S4: Partial enlargement of ^13^C NMR of CTPPPPD; Figure S5: The total ion chromatogram in positive ion mode of compounds of CTPPPPD; Figure S6: The total ion chromatogram in negative ion mode of compounds of CTPPPPD; Figure S7: OPLS-DA score plot for the CTPPPPD and Control groups in positive ion mode and negative ion mode respectively; Figure S8: Enrichment of DEGs in MAPK signaling pathway; Figure S9: Enrichment of DEGs in PI3K-AKT signaling pathway; Figure S10: Enrichment of DEGs in P53 signaling pathway; Figure S11: Enrichment of DEGs and DAMs in central carbon metabolism in cancer signaling pathway; Figure S12: Enrichment of DEGs and DAMs in aminoacyl-tRNA biosynthesis signaling pathway; Figure S13: Enrichment of DEGs and DAMs in proximal tubule bicarbonate reclamation signaling pathway; Figure S14: Enrichment of DEGs and DAMs in arginine biosynthesis signaling pathway; Figure S15: Enrichment of DEGs and DAMs in alanine, aspartate and glutamate metabolism signaling pathway.
