# Supplementary material for: Integration of Transcriptomics and Metabolomics Reveals the Antitumor Mechanism of Protopanaxadiol Triphenylphosphate Derivative in Non-Small-Cell Lung Cancer
Source: Molecules. 2024 Sep 9;29(17):4275. doi: 10.3390/molecules29174275 (PMC11396780; doi:10.3390/molecules29174275)
Supplement: Supplementary file 1 [file molecules-29-04275-s001.zip › molecules-3159373-supplementary.pdf]

Integration of Transcriptomics and Metabolomics Reveals the Antitumor Mechanism of Protopanaxadiol Triphenylphosphate Derivative in Non-Small Cell Lung Cancer

Liu Han[a], Xingbo Bian[a], Xiangyu Ma[a], Ting Ren[a], Yawei Li[a], Lijing Huang[a], Zebo Tang[b], Liancong Gao[c], Sheng Chang[a*], Xin Sun[a*]

[a] *College of pharmacy, Jilin Medical University, Jilin, China*

[b] *School of Basic Medicine, Jilin Medical University, Jilin, China*

[c] *Clinical medical school, Jilin Medical University, Jilin, China*

[*] *Corresponding author: Sheng Chang, College of pharmacy, Jilin Medical University, Jilin, China changsheng-pharm@hotmail.com; Xin Sun, College of pharmacy, Jilin Medical University, Jilin, China sunxinbh@126.com*

Table S1. Sequencing data statistics.

Table S2. Results were compared with reference genomes.

Table S3. GO results of all the enriched terms of DEGs from the comparison of CTPPPPD versus Control.

Table S4. Total ion count and identification statistics

Table S5. DAMs from the comparison of CTPPPPD versus Control.

Table S6. Enrichment of KEGG metabolic pathway of DAMs from the comparison of CTPPPPD versus Control.

Table S7. Primer information of genes used for qPCR validation.

Figure S1. HRMS of CTPPPPD.

Figure S2. ^{1}H NMR of CTPPPPD.

Figure S3. ^{13}C NMR of CTPPPPD.

Figure S4. Partial enlargement of ^{13}C NMR of CTPPPPD.

Figure S5. The total ion chromatogram in positive ion mode of compounds of CTPPPPD.

Figure S6. The total ion chromatogram in negative ion mode of compounds of CTPPPPD.

Figure S7. OPLS-DA score plot for the CTPPPPD and Control groups in positive ion mode and negative ion mode respectively.

Figure S8. Enrichment of DEGs in MAPK signaling pathway.

Figure S9. Enrichment of DEGs in PI3K-AKT signaling pathway.

Figure S10. Enrichment of DEGs in P53 signaling pathway.

Figure S11. Enrichment of DEGs and DAMs in central carbon metabolism in cancer signaling pathway.

Figure S12. Enrichment of DEGs and DAMs in aminoacyl-tRNA biosynthesis signaling pathway.

Figure S13. Enrichment of DEGs and DAMs in proximal tubule bicarbonate reclamation signaling pathway.

Figure S14. Enrichment of DEGs and DAMs in arginine biosynthesis signaling pathway.

Figure S15. Enrichment of DEGs and DAMs in alanine, aspartate and glutamate metabolism signaling pathway

Table S1. Sequencing data statistics.

Sample	Raw reads	Raw bases	Clean reads	Clean bases	Error rate (%)	Q20(%)	Q30(%)	GC content (%)
control1	47095630	7111440130	46801894	6932534968	0.0261	97.67	93.09	49.16
control2	50556842	7634083142	50180150	7439611044	0.0268	97.37	92.44	49.27
control3	43839280	6619731280	43530994	6440313871	0.0265	97.52	92.76	48.53
control4	46529056	7025887456	46153170	6782255088	0.0259	97.74	93.34	51.14
control5	43052876	6500984276	42770278	6344718164	0.0263	97.57	92.86	50.33
control6	50464500	7620139500	50120096	7407976348	0.026	97.69	93.19	50.13
CTPPPPD1	44462786	6713880686	44167302	6551424532	0.0263	97.57	92.94	48.51
CTPPPPD2	50638190	7646366690	50280072	7442651032	0.0265	97.5	92.72	48
CTPPPPD3	61165724	9236024324	60717760	8988709156	0.0259	97.74	93.32	48.84
CTPPPPD4	46261502	6985486802	45967094	6769749113	0.0263	97.6	92.96	48.55
CTPPPPD5	47748682	7210050982	47403408	7002091715	0.0265	97.51	92.78	49.17
CTPPPPD6	41338134	6242058234	41082844	6091961735	0.0262	97.62	92.97	48.29

Table S2. Results were compared with reference genomes.

Sample	Total reads	Total mapped	Multiple mapped	Unique mapped
control1	46801894	45082429(96.33%)	1620333(3.46%)	43462096(92.86%)
control2	50180150	48184561(96.02%)	1738332(3.46%)	46446229(92.56%)
control3	43530994	41762931(95.94%)	1480791(3.4%)	40282140(92.54%)
control4	46153170	44597140(96.63%)	2023687(4.38%)	42573453(92.24%)

control5	42770278	41196091(96.32%)	1656311(3.87%)	39539780(92.45%)
control6	50120096	48366084(96.5%)	1994723(3.98%)	46371361(92.52%)
CTPPPPD1	44167302	42470063(96.16%)	1443671(3.27%)	41026392(92.89%)
CTPPPPD2	50280072	48316665(96.1%)	1619264(3.22%)	46697401(92.87%)
CTPPPPD3	60717760	58462285(96.29%)	2039366(3.36%)	56422919(92.93%)
CTPPPPD4	45967094	44170237(96.09%)	1533082(3.34%)	42637155(92.76%)
CTPPPPD5	47403408	45563787(96.12%)	1583261(3.34%)	43980526(92.78%)
CTPPPPD6	41082844	39518706(96.19%)	1321040(3.22%)	38197666(92.98%)

Table S3. GO results of all the enriched terms of DEGs from the comparison of CTPPPPD versus Control.

GO id	Term description	Term type	CTPPPPD_vs_control number	CTPPPPD_vs_control percent
GO:0001906	cell killing	biological_process	8	8/ 1112
GO:0002376	immune system process	biological_process	112	112/ 1112
GO:0065007	biological regulation	biological_process	488	488/ 1112
GO:0008152	metabolic process	biological_process	312	312/ 1112
GO:0098743	cell aggregation	biological_process	1	1/ 1112
GO:0051704	multi-organism process	biological_process	80	80/ 1112
GO:0040011	locomotion	biological_process	43	43/ 1112
GO:0022414	reproductive process	biological_process	58	58/ 1112
GO:0008283	cell population proliferation	biological_process	25	25/ 1112
GO:0071840	cellular component organization or biogenesis	biological_process	199	199/ 1112
GO:0009987	cellular process	biological_process	599	599/ 1112
GO:0032502	developmental process	biological_process	218	218/ 1112
GO:0032501	multicellular organismal process	biological_process	150	150/ 1112

GO:0040007	growth	biological_process	15	15/ 1112
GO:0048511	rhythmic process	biological_process	15	15/ 1112
GO:0051179	localization	biological_process	167	167/ 1112
GO:0022610	biological adhesion	biological_process	39	39/ 1112
GO:0007610	behavior	biological_process	20	20/ 1112
GO:0098754	detoxification	biological_process	3	3/ 1112
GO:0023052	signaling	biological_process	38	38/ 1112
GO:0050896	response to stimulus	biological_process	247	247/ 1112
GO:0031974	membrane-enclosed lumen	cellular_component	57	57/ 1112
GO:0032991	protein-containing complex	cellular_component	200	200/ 1112
GO:0044456	synapse part	cellular_component	32	32/ 1112
GO:0005623	cell	cellular_component	5	5/ 1112
GO:0044425	membrane part	cellular_component	323	323/ 1112
GO:0044421	extracellular region part	cellular_component	204	204/ 1112
GO:0044422	organelle part	cellular_component	384	384/ 1112
GO:0043226	organelle	cellular_component	458	458/ 1112
GO:0045202	synapse	cellular_component	28	28/ 1112
GO:0016020	membrane	cellular_component	326	326/ 1112
GO:0030054	cell junction	cellular_component	57	57/ 1112
GO:0005576	extracellular region	cellular_component	106	106/ 1112
GO:0009295	nucleoid	cellular_component	2	2/ 1112
GO:0044217	other organism part	cellular_component	4	4/ 1112
GO:0044464	cell part	cellular_component	709	709/ 1112
GO:0099080	supramolecular complex	cellular_component	38	38/ 1112
GO:0045182	translation regulator activity	molecular_function	6	6/ 1112

GO:0140110	transcription regulator activity	molecular_function	76	76/ 1112
GO:0005198	structural molecule activity	molecular_function	41	41/ 1112
GO:0044183	protein folding chaperone	molecular_function	3	3/ 1112
GO:0038024	cargo receptor activity	molecular_function	4	4/ 1112
GO:0016209	antioxidant activity	molecular_function	5	5/ 1112
GO:0005215	transporter activity	molecular_function	56	56/ 1112
GO:0098772	molecular function regulator	molecular_function	90	90/ 1112
GO:0005488	binding	molecular_function	606	606/ 1112
GO:0060089	molecular transducer activity	molecular_function	57	57/ 1112
GO:0003824	catalytic activity	molecular_function	253	253/ 1112

Table S4. Total ion count and identification statistics

Ion mode	All peaks	Identified metabolites	Metabolites in Library	Metabolites in KEGG
pos	2377	387	365	239
neg	2920	193	177	127

Note: Identified metabolites: the number of metabolites that are ultimately identified through primary and secondary mass spectrometry data and search databases (self-built databases, Metlin, HMDB, etc.); Metabolites in library: Annotate the number of metabolites into public databases such as HMDB and Lipidmaps; Metabolites in KEGG: Annotate the number of metabolites in KEGG database.

Table S5. DAMs from the comparison of CTPPPPD versus Control.

Metabolite	Regulate	KEGG Compound ID	Mode	Formula	VIP_pred_OPLS-DA	Fold change	P value
2,4-Dichlorophenylacetic acid	down	-	neg	C8H6Cl2O2	4.6189	-6.4675	6.89E-08
3,5-Dichloro-2-hydroxybenzenesulfonic acid	down	-	neg	C6H4Cl2O4S	3.9945	-1.0649	4.95E-07
D-myo-Inositol 1,4-bisphosphate	up	C01220	neg	C6H14O12P2	2.7081	0.5145	0.000601
Pantetheine	down	C00831	neg	C11H22N2O4S	2.2378	-0.4245	0.002286
Rosmarinic acid	up	C01850	neg	C18H16O8	2.2297	0.3654	0.000524
Phosphoribosyl formamidocarboxamide	up	C04734	neg	C10H15N4O9P	2.2019	0.4273	0.001991
Dimethyl sulfoxide	down	C11143	pos	C2H6OS	2.1615	-0.3603	4.30E-07
Monoacetyldiglyceride	up	-	pos	C11H16O10	2.1571	0.4396	0.000445
Undecylenic acid	up	C13910	pos	C11H20O2	2.1191	0.3633	1.19E-05
(+/-)-Menthyl acetate	up	C09870	pos	C12H22O2	2.1002	0.2866	1.20E-06
(10Z)-Nonadec-10-enoylcarnitine	up	-	pos	C26H49NO4	2.0909	0.354	1.79E-05
Alpha-D-Glucose 1,6-Bisphosphate	up	C01231	neg	C6H14O12P2	2.0833	0.2527	6.15E-05
PS(18:1(9Z)/0:0)	up	-	neg	C24H46NO9P	2.048	0.301	0.002524
D-Galactose 1-Phosphate	up	C00446	neg	C6H13O9P	2.0434	0.2256	0.000375
Heptadecanoyl carnitine	up	-	neg	C24H47NO4	2.0022	0.2229	7.86E-07
Inosine	up	C00294	neg	C10H12N4O5	1.9783	0.1829	0.000383
Xanthosine	up	C01762	neg	C10H12N4O6	1.9756	0.2167	1.91E-06
N-Acetyl-D-Glucosamine 6-Phosphate	up	C00357	neg	C8H16NO9P	1.9646	0.2663	0.002377
Nalmefene	up	C08027	neg	C21H25NO3	1.9418	0.2705	0.000597
Valylglutamic acid	up	-	pos	C10H18N2O5	1.9367	0.3583	0.000558
Palmitoylcarnitine	up	C02990	pos	C23H45NO4	1.9156	0.203	1.38E-05

15-Hydroxyicosanoylcarnitine	up	-	pos	C27H53NO5	1.9135	0.2553	4.76E-05
Tetradecanoylcarnitine	up	-	pos	C21H41NO4	1.912	0.2719	3.83E-05
Quinic Acid	up	C00296	neg	C7H12O6	1.8807	0.3702	0.03413
2-Hydroxyoctadecanoic acid	up	C03045	neg	C18H36O3	1.8334	0.233	0.000355
Dibutyl succinate	down	C19143	neg	C12H22O4	1.822	-0.256	0.007128
N-hydroxycadaverine	down	-	pos	C5H15N2O+	1.8054	-0.5006	0.01017
Citramalic Acid	up	C00815	neg	C5H8O5	1.8037	0.1771	8.70E-08
PE(20:1/0:0)	up	-	neg	C25H50NO7P	1.7966	0.2125	0.00037
9,10-Dihydroxystearic acid	up	C19622	neg	C18H36O4	1.7894	0.1775	9.19E-05
16-Hydroxyhexadecanoic acid	up	C18218	neg	C16H32O3	1.7817	0.1698	1.99E-05
N-Jasmonoylisoleucine	up	C18699	neg	C18H29NO4	1.7727	0.1964	1.24E-05
Morroniside	down	C17000	pos	C17H26O11	1.7694	-0.3887	0.01535
Asp Ile Glu	up	-	pos	C15H25N3O8	1.76	0.3141	0.001462
1-Linoleoyl-sn-Glycero-3-Phosphocholine	up	C04100	neg	C26H50NO7P	1.7481	0.1878	0.000321
Cetylmannoside	down	-	pos	C22H44O6	1.7397	-0.2345	0.000128
N-lactoyl-phenylalanine	up	-	neg	C12H15NO4	1.731	0.184	5.07E-05
Gentamicin C2	up	C02033	neg	C20H41N5O7	1.7202	0.1447	1.58E-05
Asp Ala Arg	down	-	pos	C13H24N6O6	1.6332	-0.255	0.00174
Dodecanoylcarnitine	up	-	neg	C19H37NO4	1.615	0.174	0.000294
LysoPC(20:4(5Z,8Z,11Z,14Z)/0:0)	up	C04230	neg	C28H50NO7P	1.6135	0.1623	0.00077
Methylone	up	C20126	neg	C11H13NO3	1.6052	0.169	8.83E-05
22-Hydroxydocosanoic acid	down	C19623	pos	C22H44O3	1.5763	-0.2633	0.03025
Propionylcarnitine	down	C03017	pos	C10H19NO4	1.5738	-0.1681	8.72E-07
Palmitic Acid	up	C00249	neg	C16H32O2	1.5685	0.1437	1.90E-05
(3E,5E)-1,3,5-octatriene	down	-	pos	C8H12	1.5521	-0.1956	7.38E-08

N-[1'-(Aminocarbonyl)-2',2'-dimethylpropyl]-1-(cyclohexylmethyl)-1h-indazole-3-carboxamide	down	-	neg	C21H30N4O2	1.5489	-0.1644	0.00292
2,4-Dichlorophenol	down	C02625	neg	C6H4Cl2O	1.5229	-0.1805	0.003797
LysoPC(16:1(9Z)/0:0)	up	C04230	neg	C24H48NO7P	1.5095	0.1345	0.000492
Alpha-Ionone	down	C12286	pos	C13H20O	1.5068	-0.1933	9.53E-06
L-Palmitoylcarnitine	up	C02990	neg	C23H45NO4	1.4952	0.1074	5.16E-05
1-Kestose	down	C03661	pos	C18H32O16	1.4928	-0.1555	0.000248
Ribose 1-phosphate	up	C00620	neg	C5H11O8P	1.4825	0.1418	0.001112
Arachidonic acid	up	C00219	neg	C20H32O2	1.4797	0.159	0.00161
THIARABINE	down	-	neg	C9H13N3O4S	1.4633	-0.1502	0.002049
L-a-Lysophosphatidylserine	up	-	neg	C24H48NO9P	1.4576	0.1365	0.000367
N-Oleoyl Glutamic acid	down	-	pos	C23H41NO5	1.4502	-0.1512	5.67E-06
UDP-glucose	up	C00029	neg	C15H24N2O17P2	1.441	0.1114	0.001496
19-Hydroxyprostaglandin E1	down	-	pos	C20H34O6	1.4361	-0.1432	1.30E-06
Maltotriose	down	C01835	neg	C18H32O16	1.432	-0.1264	0.001262
Daucol	down	C09652	pos	C15H26O2	1.431	-0.1605	4.91E-05
AB-Chminaca	down	-	neg	C20H28N4O2	1.4301	-0.1419	0.002512
Pseudouridine	up	C02067	neg	C9H12N2O6	1.4301	0.1104	0.000135
Gamma-Glutamylisoleucine	up	-	neg	C11H20N2O5	1.4127	0.1264	0.000107
Behenic acid	down	C08281	pos	C22H44O2	1.397	-0.1811	0.03197
N-Acetylaspartylglutamic Acid	up	C12270	neg	C11H16N2O8	1.3924	0.1043	0.000234
Gamma-Glutamylleucine	up	-	neg	C11H20N2O5	1.3906	0.1308	0.000161
Arabinosylhypoxanthine	up	-	pos	C10H12N4O5	1.3901	0.126	0.001897
PE(18:2/0:0)	up	-	neg	C23H44NO7P	1.3884	0.1342	0.00298
15-Deoxyprostaglandin J2	down	C14717	pos	C20H30O3	1.3878	-0.2127	0.02909
4-(2,6,6-Trimethyl-1-cyclohexenyl)-2-butanol	up	-	neg	C13H24O	1.3643	0.1341	0.000203

PE(16:1/0:0)	up	-	neg	C21H42NO7P	1.3583	0.1197	0.001083
N6-Succinyl Adenosine	up	-	neg	C14H17N5O8	1.3523	0.1193	0.007264
Coformycin	up	C01677	pos	C11H16N4O5	1.3436	0.2026	0.01622
LysoPG(18:1(9Z)/0:0)	up	-	neg	C24H47O9P	1.3376	0.1185	0.004086
5-Methyl-THF	up	-	neg	C20H25N7O6	1.3043	0.1421	0.02871
O-Acetotoluidide	down	-	pos	C9H11NO	1.2942	-0.1514	6.78E-05
MG(0:0/i-15:0/0:0)	down	-	pos	C18H36O4	1.2912	-0.1397	0.00066
Demethylphylloquinone	up	C13309	neg	C30H44O2	1.2891	0.0986	0.000104
5(S)-HETE	up	C04805	neg	C20H32O3	1.2874	0.111	0.000327
N-Acetylneuraminic Acid	up	C00270	neg	C11H19NO9	1.2873	0.1158	0.001989
4-Amino-1-butanol	down	-	pos	C4H11NO	1.2812	-0.1383	8.75E-06
Uridine diphosphate-N-acetylglucosamine	up	C00043	neg	C17H27N3O17P2	1.28	0.0837	0.002143
Ecgonine	up	C10858	pos	C9H15NO3	1.2783	0.1444	0.000594
N-Acetylmuramate	up	C02713	pos	C11H19NO8	1.2769	0.1555	0.002031
Aceteugenol	down	C14567	pos	C12H14O3	1.2709	-0.1123	2.44E-07
LysoPE(P-18:0/0:0)	up	-	neg	C23H48NO6P	1.269	0.096	0.001533
LysoPE(0:0/18:0)	down	-	pos	C23H48NO7P	1.2651	-0.0993	6.84E-06
Hymenoxon	down	C09482	pos	C15H22O5	1.259	-0.0977	2.80E-08
8,11-eicosadiynoic acid	up	-	neg	C20H32O2	1.2538	0.1387	0.01247
4-Methylumbelliferone	down	C03081	pos	C10H8O3	1.2489	-0.1215	7.40E-07
Alpha-AMINO-3-HYDROXY-5-METHYL-4-ISOXAZOLEPROPIONIC ACID	up	C13672	neg	C7H10N2O4	1.2349	0.1158	0.002046
Glycerol 3-Phosphate	up	C00093	neg	C3H9O6P	1.2264	0.1457	0.04362
Uridine	up	C00299	neg	C9H12N2O6	1.2252	0.1192	0.01006
PE(18:1/0:0)	up	-	neg	C23H46NO7P	1.2222	0.0792	0.001094
Myristoylglycine	down	-	pos	C16H31NO3	1.22	-0.1167	6.90E-05

N,N-Dimethylaniline	down	C02846	pos	C8H11N	1.2156	-0.1072	1.35E-05
Abscisic alcohol	down	C13456	pos	C15H22O3	1.2154	-0.1151	0.000127
Salviol	down	C21819	pos	C20H30O2	1.2134	-0.1087	2.59E-06
Leukotriene E3	down	-	pos	C23H39NO5S	1.2068	-0.0705	3.14E-06
Glycylproline	up	-	pos	C7H12N2O3	1.1962	0.152	0.003941
1,2,4-Benzotriazin-3-amine 1-oxide	down	-	pos	C7H6N4O	1.1931	-0.0982	9.55E-08
UDP-D-Galactose	up	C00052	neg	C15H24N2O17P2	1.1929	0.077	0.001318
2-Methylaniline	down	C14403	pos	C7H9N	1.1918	-0.094	7.41E-08
6-Methylpteridine-2,4-diamine	down	-	pos	C7H8N6	1.1911	-0.0882	2.06E-07
Azacyclotridecan-2-one	down	-	pos	C12H23NO	1.182	-0.1038	2.07E-05
Mimosine	up	C04771	neg	C8H10N2O4	1.1802	0.0775	0.000679
LysoPC(0:0/18:2(9Z,12Z))	up	-	pos	C26H50NO7P	1.1795	0.1057	0.004963
Octanoate	down	C06423	pos	C8H15O2-	1.1772	-0.086	2.29E-07
Menthyl ethylene glycol carbonate	down	-	pos	C13H24O4	1.177	-0.0787	1.09E-07
Alpha-Bisabolol oxide A	down	C16773	pos	C15H26O2	1.1747	-0.103	4.57E-06
1-O-Isopentyl-3-O-octadec-2-enoyl glycerol	down	-	pos	C26H50O4	1.167	-0.0616	1.74E-07
2-Phenylethanol glucuronide	down	C03033	pos	C14H18O7	1.1641	-0.1097	0.000364
Tiglic acid	down	C08279	pos	C5H8O2	1.1634	-0.1278	1.27E-05
8-Hydroxy-5,6-octadienoic acid	down	-	pos	C8H12O3	1.1632	-0.1499	0.01411
Floionolic acid	down	C19621	pos	C18H36O5	1.1619	-0.079	2.18E-07
7,8-diaminopelargonate	down	C01037	pos	C9H20N2O2	1.1612	-0.1014	3.64E-06
Beta-D-Glucopyranosyl anthranilate	up	-	pos	C13H17NO7	1.1581	0.1413	0.02701
Methenamine	down	-	pos	C6H12N4	1.158	-0.0827	1.90E-08
Metazin	down	-	pos	C15H30N6O6	1.1566	-0.0773	7.81E-08
Vomifoliol	down	C04166	pos	C13H20O3	1.1528	-0.12	0.000263
Harmalol	down	C06537	pos	C12H12N2O	1.152	-0.105	0.004083

Carbamoyl (2R)-2,5-diaminopentanoate	up	-	neg	C6H13N3O3	1.1502	0.0698	3.26E-05
PA(i-14:0/i-13:0)	down	C00416	pos	C30H59O8P	1.1476	-0.107	0.00073
(3Z,6Z)-3,6-Nonadienal	down	C16323	pos	C9H14O	1.1451	-0.0764	1.53E-07
5,7-Megastigmadien-9-ol glucoside	down	-	pos	C19H32O6	1.1444	-0.0833	4.21E-06
N-Undecanoylglycine	down	-	pos	C13H25NO3	1.1436	-0.0792	2.65E-05
Exemestane	down	C08162	pos	C20H24O2	1.1416	-0.0888	1.03E-06
1,2-Dihydronaphthalene	down	-	pos	C10H10	1.1412	-0.12	0.000102
D-Ornithine	up	C00515	neg	C5H12N2O2	1.1407	0.072	4.41E-05
(11R,16S)-misoprostol	down	-	pos	C22H38O5	1.1399	-0.081	1.57E-07
Tetradecanedioic acid	up	C11002	neg	C14H26O4	1.1385	0.1112	0.004367
Alpha-Campholonic acid	down	-	pos	C10H16O3	1.1374	-0.0834	7.91E-07
L-Tryptophan	up	C00078	neg	C11H12N2O2	1.1295	0.0822	0.00118
NeuNGc	down	C03410	pos	C11H19NO10	1.1276	-0.1482	0.02438
N-Docosahexaenoyl Arginine	down	-	pos	C28H44N4O3	1.1275	-0.0838	1.14E-06
KAPA	down	C01092	pos	C9H17NO3	1.1271	-0.1162	0.01512
O-Toluidine	down	C14403	pos	C7H9N	1.1254	-0.0862	1.52E-07
4-Hydroxynonenal	up	C21642	pos	C9H16O2	1.1251	0.1134	0.001993
(Z)-5-[(2R,3S,4S)-4-Hydroxy-2-[(E)-3-hydroxyoct-1-enyl]oxan-3-yl]pent-3-enoic acid	down	-	pos	C18H30O5	1.121	-0.1092	0.000984
Morpholine	down	C14452	pos	C4H9NO	1.1191	-0.1045	1.32E-06
Diethanolamine	down	C06772	pos	C4H11NO2	1.1184	-0.0996	1.88E-07
Ethyl trans-p-methoxycinnamate	down	C10476	pos	C12H14O3	1.1182	-0.0893	9.77E-06
1-Methyladenosine	up	C02494	pos	C11H15N5O4	1.1149	0.1297	0.002144
L-quinate	down	C00296	pos	C7H11O6-	1.111	-0.09	1.33E-06
(E,E)-2,4-Hexadienal	down	C19249	pos	C6H8O	1.1108	-0.0885	3.87E-07
Lubiprostone	down	C13707	pos	C20H32F2O5	1.1073	-0.0738	3.30E-07

5-Methoxyindoleacetate	up	C05660	pos	C11H11NO3	1.1068	0.1221	0.004977
13(S)-Hydroperoxylinolenic acid	down	C04785	pos	C18H30O4	1.1057	-0.0902	8.48E-06
Deoxyinosine	up	C05512	neg	C10H12N4O4	1.1029	0.0984	0.01247
Cinncassiol D3	down	C17655	pos	C20H32O6	1.1022	-0.0735	9.33E-07
MJDBISSP00000002	down	-	pos	C23H42D3NO4	1.1019	-0.0643	3.64E-06
Oleoyl-L-Carnitine	up	-	pos	C25H47NO4	1.101	0.0853	0.01885
L-Glutamic Acid	up	C00025	neg	C5H9NO4	1.1001	0.0647	0.002389
L-Isoleucine	down	C00407	pos	C6H13NO2	1.0953	-0.0988	0.000113
N,N,N',N',N'',N''-Hexakis(methoxymethyl)-1,3,5-triazine-2,4,6-triamine	down	-	pos	C15H30N6O6	1.0945	-0.0696	1.05E-06
Quassin	down	C17029	pos	C22H28O6	1.0937	-0.0818	0.002
Octyl hydrogen phthalate	up	-	neg	C16H22O4	1.0902	0.082	0.002133
Tanacetol B	down	-	pos	C17H28O4	1.0897	-0.0734	1.32E-06
Alpha-Methylene-Gamma-Butyrolactone	down	C20578	pos	C5H6O2	1.0867	-0.072	3.37E-07
Prostaglandin F1a	down	C06475	pos	C20H36O5	1.0865	-0.0763	3.73E-07
Stearyl citrate	down	-	pos	C24H44O7	1.0838	-0.0936	0.000719
Tranexamic Acid	down	C12535	pos	C8H15NO2	1.0838	-0.1039	0.001802
LysoPC(14:0/0:0)	up	C04230	pos	C22H46NO7P	1.0787	0.0961	0.003639
O-Xylene	down	C07212	pos	C8H10	1.0759	-0.1618	0.02237
Chembl4238926	down	-	pos	C18H22N4O2	1.0708	-0.0691	1.56E-06
Hexadienic acid	down	-	pos	C6H8O2	1.0686	-0.0923	2.32E-06
Dihydrozeatin	down	C02029	pos	C10H15N5O	1.0684	-0.0825	4.92E-06
13,16,19-Docosatrienoic acid	down	C16534	pos	C22H38O2	1.0661	-0.108	0.00275
1H-Pyrazolo[3,4-d]pyrimidin-4-amine	down	-	pos	C5H5N5	1.0626	-0.0713	3.14E-06
(6R,7S)-6,7-Epoxy-1,3-tetradecadiyne	down	-	pos	C14H20O	1.0561	-0.0669	7.81E-07
Monopropionylcadaverine	down	-	pos	C8H18N2O	1.0554	-0.0763	1.31E-05

L-beta-aspartyl-L-leucine	up	-	neg	C10H18N2O5	1.0531	0.0757	0.001916
2-Aminopurine	down	-	pos	C5H5N5	1.0489	-0.0691	1.08E-06
L-Glutamine	up	C00064	neg	C5H10N2O3	1.0487	0.0587	0.001567
Triethanolamine	down	C06771	pos	C6H15NO3	1.0487	-0.0725	1.49E-06
UDP-N-acetyl-D-mannosamine	up	C01170	neg	C17H27N3O17P2	1.0467	0.0573	0.003511
(2R,3R,4R)-2-Amino-4-hydroxy-3-methylpentanoic acid	down	-	pos	C6H13NO3	1.045	-0.0654	1.02E-06
N-(5-methyl-1,2-oxazol-3-yl)pyrimidine-2-carboxamide	down	-	pos	C9H8N4O2	1.045	-0.0742	2.48E-07
Statine	down	-	pos	C8H17NO3	1.0393	-0.0818	2.88E-06
Pipecolic Acid	down	C00408	pos	C6H11NO2	1.0372	-0.0769	7.57E-06
1-[(2R,3S,5R)-3,4-Dihydroxy-5-(hydroxymethyl)oxolan-2-yl]pyrimidine-2,4-dione	up	-	neg	C9H12N2O6	1.0362	0.0934	0.03714
GDP-Glucose	up	C00394	neg	C16H25N5O16P2	1.0356	0.0899	0.03105
9-Ethylguanine	down	-	pos	C7H9N5O	1.0346	-0.0776	1.57E-06
MJDBISSP00000009	down	-	pos	C9H6D5NO2	1.0309	-0.0526	1.78E-06
Icariside B8	down	-	pos	C19H32O8	1.0234	-0.0798	0.00019
Benzenebutanoic acid, alpha-(acetylamino)-2-amino-gamma-oxo-	up	-	neg	C12H14N2O4	1.0208	0.0764	0.004354
Beta-Alanyl-L-lysine	down	C05341	pos	C9H19N3O3	1.0206	-0.0615	3.33E-06
L-Carnitine	down	C00318	pos	C7H15NO3	1.0193	-0.0676	4.66E-05
LysoPE(0:0/20:1(11Z))	up	-	neg	C25H50NO7P	1.0189	0.0994	0.03324
Benzaldehyde	down	C00261	pos	C7H6O	1.0181	-0.0781	6.57E-07
Glycerylphosphorylcholine	up	C00670	pos	C8H20NO6P	1.017	0.0809	0.001306
L-Aspartic Acid	down	C00049	pos	C4H7NO4	1.0165	-0.0776	0.000969
Furfural	down	C14279	pos	C5H4O2	1.0159	-0.089	2.52E-06
Diisobutyl phthalate	down	C15205	pos	C16H22O4	1.0157	-0.0787	6.35E-05
Umbelliferone	down	C09315	pos	C9H6O3	1.0146	-0.0669	3.97E-07

LysoPC(20:4(8Z,11Z,14Z,17Z)/0:0)	up	C04230	pos	C28H50NO7P	1.014	0.0869	0.01633
Cucurbic acid	down	C08482	pos	C12H20O3	1.0118	-0.0622	4.89E-06
2-Methylbutyroylcarnitine	down	-	pos	C12H23NO4	1.0114	-0.1047	0.006731
MJDBISSP00000001	down	-	pos	C9H10ClNO2	1.0054	-0.045	1.59E-06
METHACHOLINE	down	C07471	pos	C8H17NO2	1.0054	-0.0864	0.000214
Maltol	down	C11918	pos	C6H6O3	1.0046	-0.0607	9.00E-07
UDP-L-iduronate	up	C02330	neg	C15H22N2O18P2	1.0017	0.0592	0.001312
Glutamylleucine	up	-	pos	C11H20N2O5	1.0006	0.112	0.02085

Table S6. Enrichment of KEGG metabolic pathway of DAMs from the comparison of CTPPPPD versus Control.

Number	First Category	Second Category	Pathway ID	Pathway Description	Impact value	P value
1	Human Diseases	Cancer: specific types	map05212	Pancreatic cancer	0.5	0.0284
1	Environmental Information Processing	Signal transduction	map04068	FoxO signaling pathway	0.2	0.0695
1	Human Diseases	Substance dependence	map05033	Nicotine addiction	0.142857	0.096
1	Human Diseases	Neurodegenerative disease	map05017	Spinocerebellar ataxia	0.142857	0.096
1	Human Diseases	Substance dependence	map05030	Cocaine addiction	0.142857	0.096
1	Organismal Systems	Nervous system	map04720	Long-term potentiation	0.142857	0.096
1	Human Diseases	Neurodegenerative disease	map05016	Huntington disease	0.166667	0.0828
1	Human Diseases	Infectious disease: parasitic	map05140	Leishmaniasis	0.166667	0.0828
1	Human Diseases	Infectious disease: parasitic	map05143	African trypanosomiasis	0.125	0.1089
1	Human Diseases	Substance dependence	map05031	Amphetamine addiction	0.111111	0.1217
1	Organismal Systems	Environmental adaptation	map04713	Circadian entrainment	0.111111	0.1217
1	Cellular Processes	Cell growth and death	map04217	Necroptosis	0.1	0.1343

1	Human Diseases	Substance dependence	map05034	Alcoholism	0.1	0.1343
1	Cellular Processes	Cellular community - eukaryotes	map04540	Gap junction	0.090909	0.1467
1	Organismal Systems	Immune system	map04664	Fc epsilon RI signaling pathway	0.090909	0.1467
1	Organismal Systems	Endocrine system	map04921	Oxytocin signaling pathway	0.083333	0.1589
1	Organismal Systems	Nervous system	map04721	Synaptic vesicle cycle	0.083333	0.1589
1	Human Diseases	Infectious disease: parasitic	map05146	Amoebiasis	0.076923	0.171
1	Metabolism	Metabolism of cofactors and vitamins	map00785	Lipoic acid metabolism	0.076923	0.171
1	Organismal Systems	Digestive system	map04975	Fat digestion and absorption	0.076923	0.171
1	Organismal Systems	Endocrine system	map04923	Regulation of lipolysis in adipocytes	0.071429	0.1829
1	Human Diseases	Neurodegenerative disease	map05014	Amyotrophic lateral sclerosis	0.071429	0.1829
1	Human Diseases	Infectious disease: bacterial	map05131	Shigellosis	0.071429	0.1829
1	Human Diseases	Cancer: specific types	map05225	Hepatocellular carcinoma	0.071429	0.1829
1	Organismal Systems	Immune system	map04611	Platelet activation	0.071429	0.1829
1	Organismal Systems	Circulatory system	map04270	Vascular smooth muscle contraction	0.0625	0.2061
1	Human Diseases	Endocrine and metabolic disease	map04931	Insulin resistance	0.052632	0.2398
1	Metabolism	Amino acid metabolism	map00290	Valine, leucine and isoleucine biosynthesis	0.043478	0.2826
1	Organismal Systems	Environmental adaptation	map04714	Thermogenesis	0.043478	0.2826
1	Organismal Systems	Endocrine system	map04925	Aldosterone synthesis and secretion	0.045455	0.2721
1	Metabolism	Biosynthesis of other secondary metabolites	map00232	Caffeine metabolism	0.045455	0.2721
1	Metabolism	Metabolism of other amino acids	map00430	Taurine and hypotaurine metabolism	0.045455	0.2721
1	Organismal Systems	Endocrine system	map04913	Ovarian steroidogenesis	0.041667	0.2929
1	Environmental Information Processing	Signal transduction	map04024	cAMP signaling pathway	0.04	0.3031
1	Metabolism	Lipid metabolism	map00565	Ether lipid metabolism	0.04	0.3031
1	Organismal Systems	Digestive system	map04973	Carbohydrate digestion and absorption	0.037037	0.323
1	Metabolism	Lipid metabolism	map00591	Linoleic acid metabolism	0.035714	0.3327

1	Metabolism	Metabolism of cofactors and vitamins	map00750	Vitamin B6 metabolism	0.034483	0.3423
1	Organismal Systems	Sensory system	map04742	Taste transduction	0.03125	0.3703
1	Human Diseases	Neurodegenerative disease	map05022	Pathways of neurodegeneration - multiple diseases	0.03125	0.3703
1	Metabolism	Energy metabolism	map00920	Sulfur metabolism	0.030303	0.3794
1	Metabolism	Carbohydrate metabolism	map00030	Pentose phosphate pathway	0.028571	0.3972
1	Metabolism	Metabolism of other amino acids	map00480	Glutathione metabolism	0.026316	0.4229
1	Metabolism	Lipid metabolism	map00062	Fatty acid elongation	0.025	0.4394
1	Metabolism	Carbohydrate metabolism	map00650	Butanoate metabolism	0.02381	0.4555
1	Metabolism	Amino acid metabolism	map00280	Valine, leucine and isoleucine degradation	0.02381	0.4555
1	Metabolism	Carbohydrate metabolism	map00562	Inositol phosphate metabolism	0.021277	0.4937
1	Metabolism	Amino acid metabolism	map00310	Lysine degradation	0.02	0.5153
1	Metabolism	Metabolism of cofactors and vitamins	map00760	Nicotinate and nicotinamide metabolism	0.018182	0.5494
1	Metabolism	Amino acid metabolism	map00270	Cysteine and methionine metabolism	0.015625	0.6049
1	Metabolism	Metabolism of cofactors and vitamins	map00130	Ubiquinone and other terpenoid-quinone biosynthesis	0.014085	0.6434
1	Metabolism	Amino acid metabolism	map00350	Tyrosine metabolism	0.012821	0.6782
1	Metabolism	Amino acid metabolism	map00330	Arginine and proline metabolism	0.012821	0.6782
1	Metabolism	Metabolism of cofactors and vitamins	map00860	Porphyrin and chlorophyll metabolism	0.006944	0.8787
2	Organismal Systems	Endocrine system	map04912	GnRH signaling pathway	0.333333	0.0029
2	Organismal Systems	Immune system	map04666	Fc gamma R-mediated phagocytosis	0.25	0.0053
2	Organismal Systems	Nervous system	map04724	Glutamatergic synapse	0.25	0.0053
2	Organismal Systems	Nervous system	map04727	GABAergic synapse	0.222222	0.0068
2	Organismal Systems	Nervous system	map04730	Long-term depression	0.222222	0.0068
2	Environmental Information Processing	Signal transduction	map04072	Phospholipase D signaling pathway	0.181818	0.0102

2	Organismal Systems	Excretory system	map04964	Proximal tubule bicarbonate reclamation	0.117647	0.0238
2	Organismal Systems	Nervous system	map04723	Retrograde endocannabinoid signaling	0.105263	0.0294
2	Metabolism	Energy metabolism	map00910	Nitrogen metabolism	0.105263	0.0294
2	Metabolism	Metabolism of cofactors and vitamins	map00780	Biotin metabolism	0.071429	0.0599
2	Human Diseases	Cancer: overview	map05200	Pathways in cancer	0.064516	0.0718
2	Metabolism	Metabolism of cofactors and vitamins	map00770	Pantothenate and CoA biosynthesis	0.066667	0.0677
2	Environmental Information Processing	Signal transduction	map04070	Phosphatidylinositol signaling system	0.068966	0.0638
2	Cellular Processes	Cell growth and death	map04216	Ferroptosis	0.068966	0.0638
2	Metabolism	Metabolism of other amino acids	map00410	beta-Alanine metabolism	0.0625	0.0759
2	Organismal Systems	Sensory system	map04750	Inflammatory mediator regulation of TRP channels	0.057143	0.0886
2	Human Diseases	Cardiovascular disease	map05415	Diabetic cardiomyopathy	0.051282	0.1065
2	Organismal Systems	Nervous system	map04726	Serotonergic synapse	0.047619	0.1204
2	Metabolism	Lipid metabolism	map00592	alpha-Linolenic acid metabolism	0.045455	0.13
2	Metabolism	Amino acid metabolism	map00340	Histidine metabolism	0.042553	0.1447
2	Metabolism	Amino acid metabolism	map00260	Glycine, serine and threonine metabolism	0.04	0.1597
2	Metabolism	Carbohydrate metabolism	map00053	Ascorbate and aldarate metabolism	0.035088	0.1956
2	Metabolism	Lipid metabolism	map00061	Fatty acid biosynthesis	0.034483	0.2009
2	Metabolism	Carbohydrate metabolism	map00040	Pentose and glucuronate interconversions	0.034483	0.2009
2	Metabolism	Carbohydrate metabolism	map00630	Glyoxylate and dicarboxylate metabolism	0.032258	0.2219
2	Metabolism	Amino acid metabolism	map00380	Tryptophan metabolism	0.024096	0.3337
2	Organismal Systems	Digestive system	map04976	Bile secretion	0.020619	0.4061
3	Metabolism	Amino acid metabolism	map00220	Arginine biosynthesis	0.130435	0.004
3	Organismal Systems	Digestive system	map04978	Mineral absorption	0.103448	0.0078
3	Metabolism	Lipid metabolism	map00561	Glycerolipid metabolism	0.078947	0.0165

3	Metabolism	Carbohydrate metabolism	map00500	Starch and sucrose metabolism	0.081081	0.0153
3	Metabolism	Carbohydrate metabolism	map00052	Galactose metabolism	0.065217	0.0273
3	Environmental Information Processing	Signaling molecules and interaction	map04080	Neuroactive ligand-receptor interaction	0.057692	0.0375
3	Metabolism	Amino acid metabolism	map00400	Phenylalanine, tyrosine and tryptophan biosynthesis	0.058824	0.0843
3	Metabolism	Lipid metabolism	map00590	Arachidonic acid metabolism	0.04	0.0911
3	Metabolism	Lipid metabolism	map01040	Biosynthesis of unsaturated fatty acids	0.040541	0.0883
3	Metabolism	Lipid metabolism	map00071	Fatty acid degradation	0.04	0.1597
4	Metabolism	Amino acid metabolism	map00250	Alanine, aspartate and glutamate metabolism	0.142857	0.0006
4	Metabolism	Nucleotide metabolism	map00240	Pyrimidine metabolism	0.061538	0.0134
4	Metabolism	Biosynthesis of other secondary metabolites	map00524	Neomycin, kanamycin and gentamicin biosynthesis	0.049383	0.0278
5	Human Diseases	Cancer: overview	map05230	Central carbon metabolism in cancer	0.135135	0.0002
5	Organismal Systems	Digestive system	map04974	Protein digestion and absorption	0.106383	0.0005
5	Genetic Information Processing	Translation	map00970	Aminoacyl-tRNA biosynthesis	0.096154	0.0008
6	Metabolism	Nucleotide metabolism	map00230	Purine metabolism	0.063158	0.0022
7	Human Diseases	Cancer: overview	map05231	Choline metabolism in cancer	0.363636	0
9	Metabolism	Lipid metabolism	map00564	Glycerophospholipid metabolism	0.107143	0.0001
10	Environmental Information Processing	Membrane transport	map02010	ABC transporters	0.072993	0
10	Metabolism	Global and overview maps	map01240	Biosynthesis of cofactors	0.030581	0.0163
11	Metabolism	Carbohydrate metabolism	map00520	Amino sugar and nucleotide sugar metabolism	0.101852	0

Table S7. Primer information of genes used for qPCR validation.

Gene	Sequence (5' – 3')	Forward/Reverse
PCK2	GGTCTTTGCTCTCTCGGGTTTA	F
	CGTCCTGTTGTCTGTCCTCTCAT	R
ITGA5	GGAGGGAGCGTTTGAAGAAT	F
	GCAGGATGTAGAGGTTGGAGG	R
THBS1	ATCCCTGAGGCAGATGAAGAAG	F
	ATTGGACACCACGCTGAAGAC	R
FGFR3	GCCGTGTTTGTACTTTGCTCTT	F
	GGTGGGCAAGGTTCTGTATCC	R
TGFB1	CCTCGAGAAGGCAAGAAAACA	F
	CACCCATCCTCCCCAACAGTA	R
DDIT3	CCTGCTTCTCTGGCTTGGCT	F
	CCGTTTCCTGGTTCTCCCTT	R
GADD45A	CCCGATAACGTGGTGTTGTG	F
	CGCCTGGATCAGGGTGAAGT	R
GTSE1	TTTCACCTCTTCCAGCCACTC	F
	CACAACAGACCACATCCTTCG	R
SESN2	ATGGCACCACTCACTCACACC	F
	TACGGAAATACAGCCCAAGCA	R
PGAM1	CAGTCTGCTCTTTGTGGTGTTG	F
	TGTGAATGGCTGCTTCTTTGTC	R
SLC2A1	TCAGTTTCCCCAGGGTCCA	F
	CATTCTCAAAGCCTTCCAGGC	R

LDHA	GCCCATAGAGCCAAAAAAGC	F
	TCCCAAGAGAAAAATGCAAGC	R

Qualitative Analysis Report

**Data Filename**	P8.d	**Sample Name**	P8
**Sample Type**	Sample	**Position**	P1-F3
**Instrument Name**	Instrument 1	**User Name**	
**Acq Method**	0-1400 pos.m	**Acquired Time**	9/18/2020 10:55:31 PM
**IRM Calibration Status**	Success	**DA Method**	3.m
**Comment**			

Sample Group **Info.**

Acquisition SW 6200 series TOF/6500 series

Version Q-TOF B.05.01 (B5125)

User Spectra

Fragmentor Voltage	Collision Energy	Ionization Mode
100	0	ESI

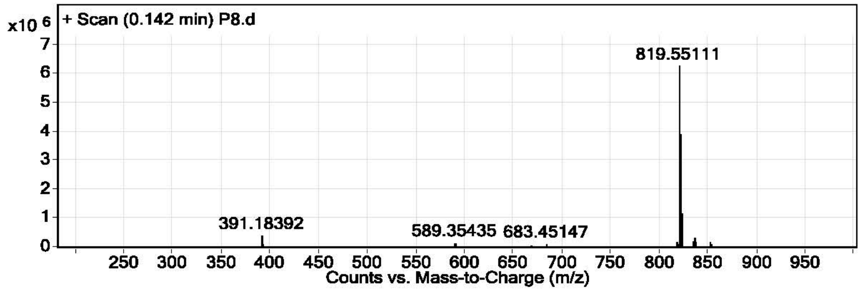

Figure S1. HRMS of CTPPPPD.

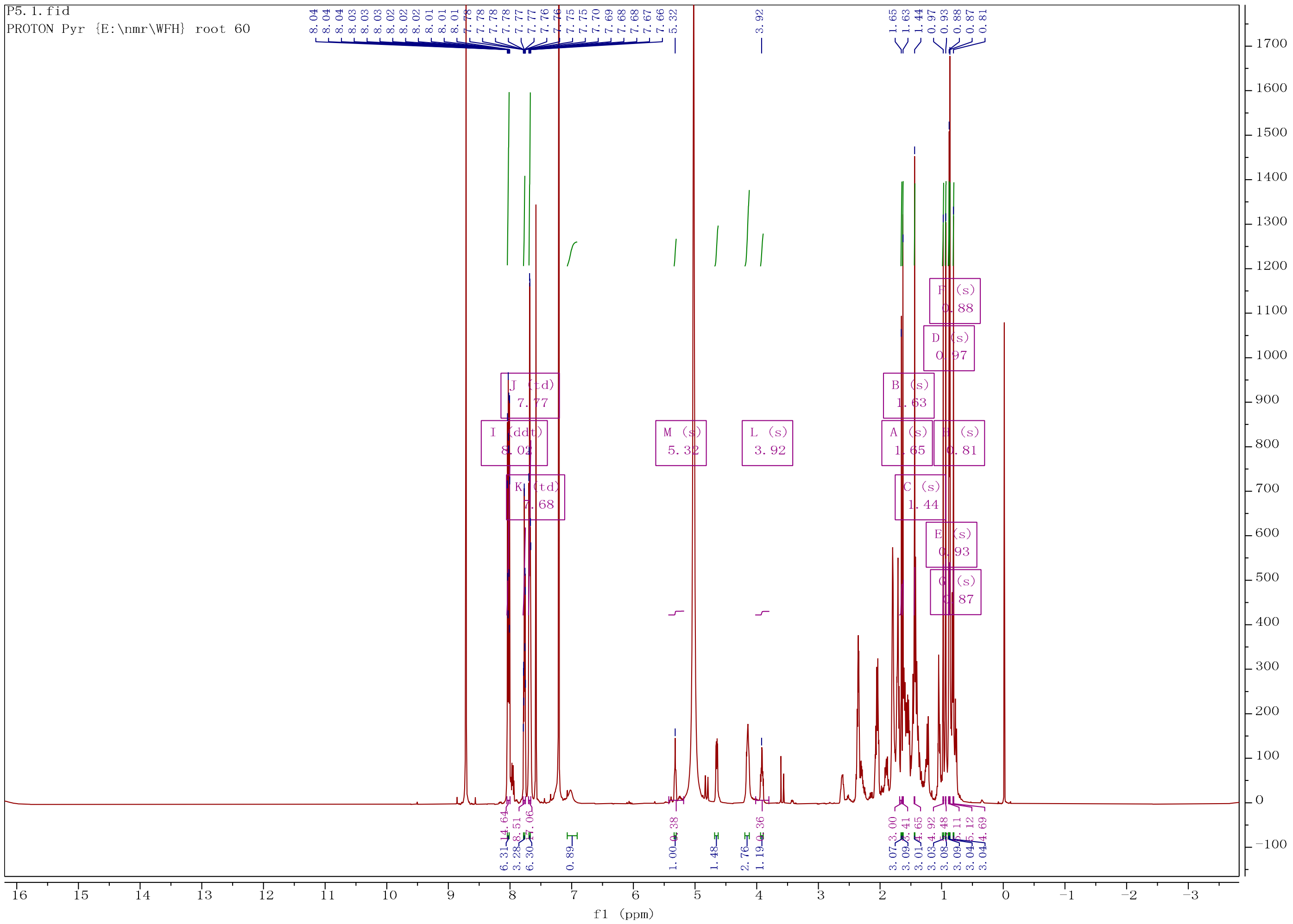

Figure S2. ^{1}H NMR of CTPPPPD.

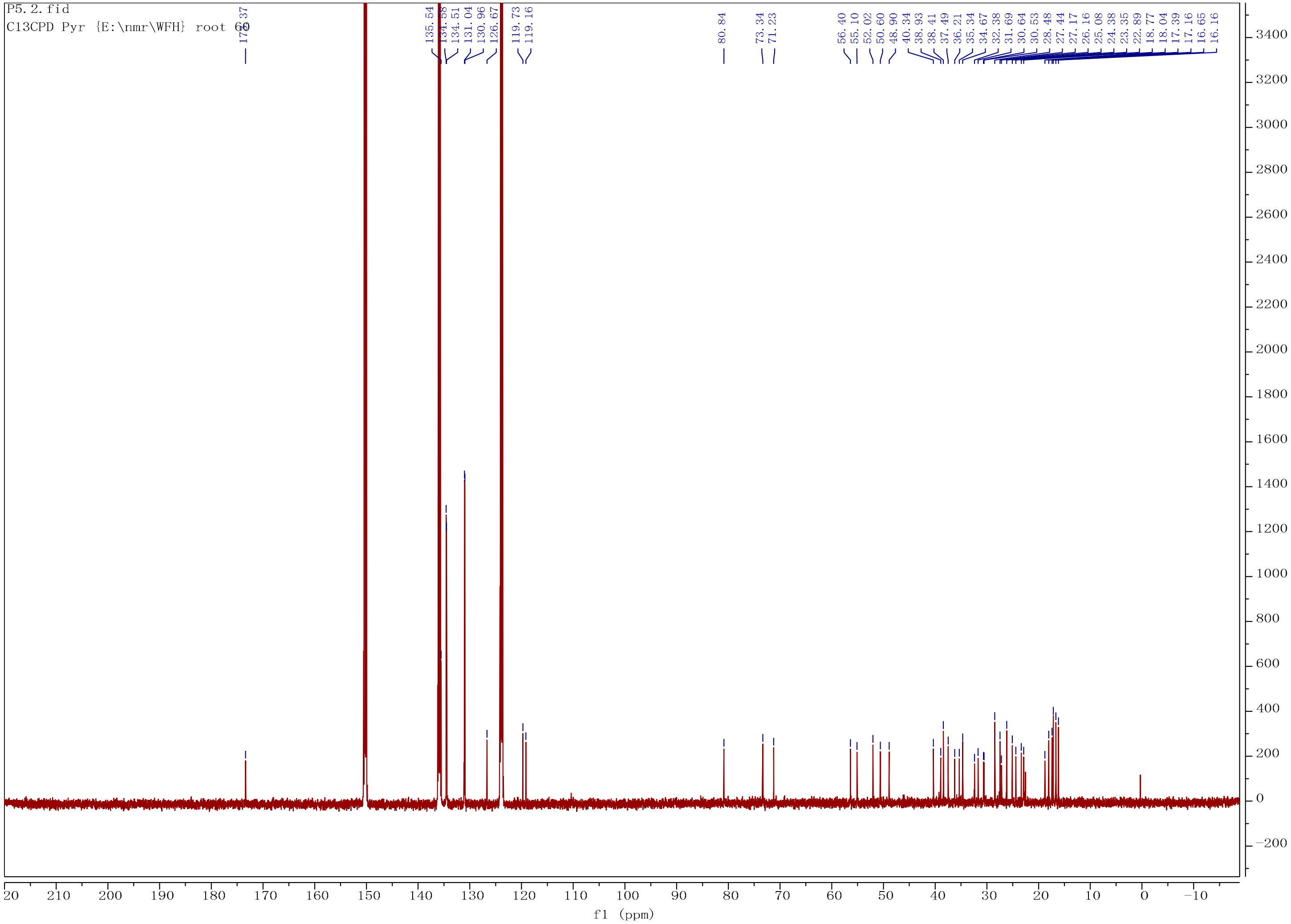

Figure S3. ^{13}C NMR of CTPPPPD.

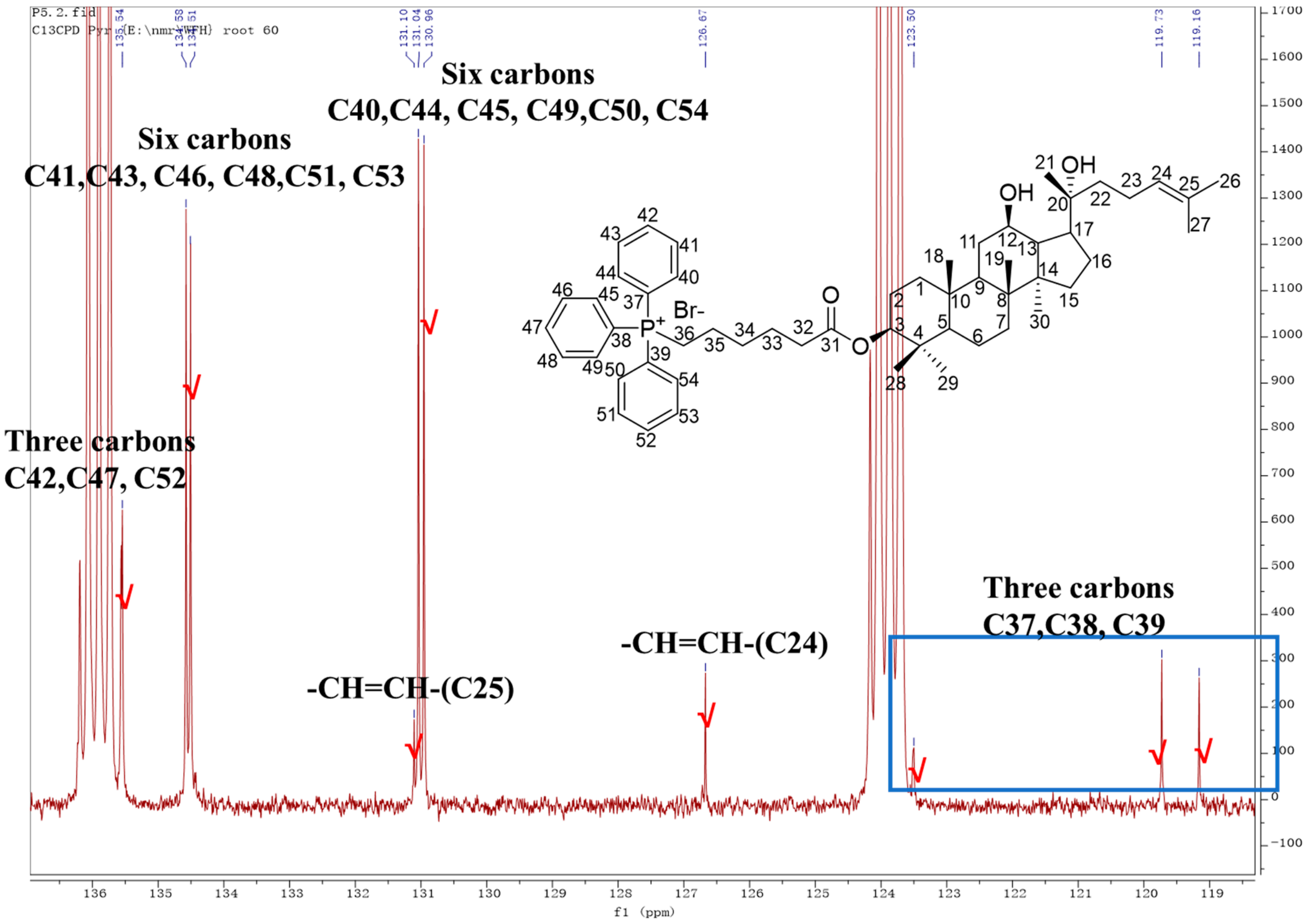

Figure S4. Partial enlargement of ^{13}C NMR of CTPPPPD.

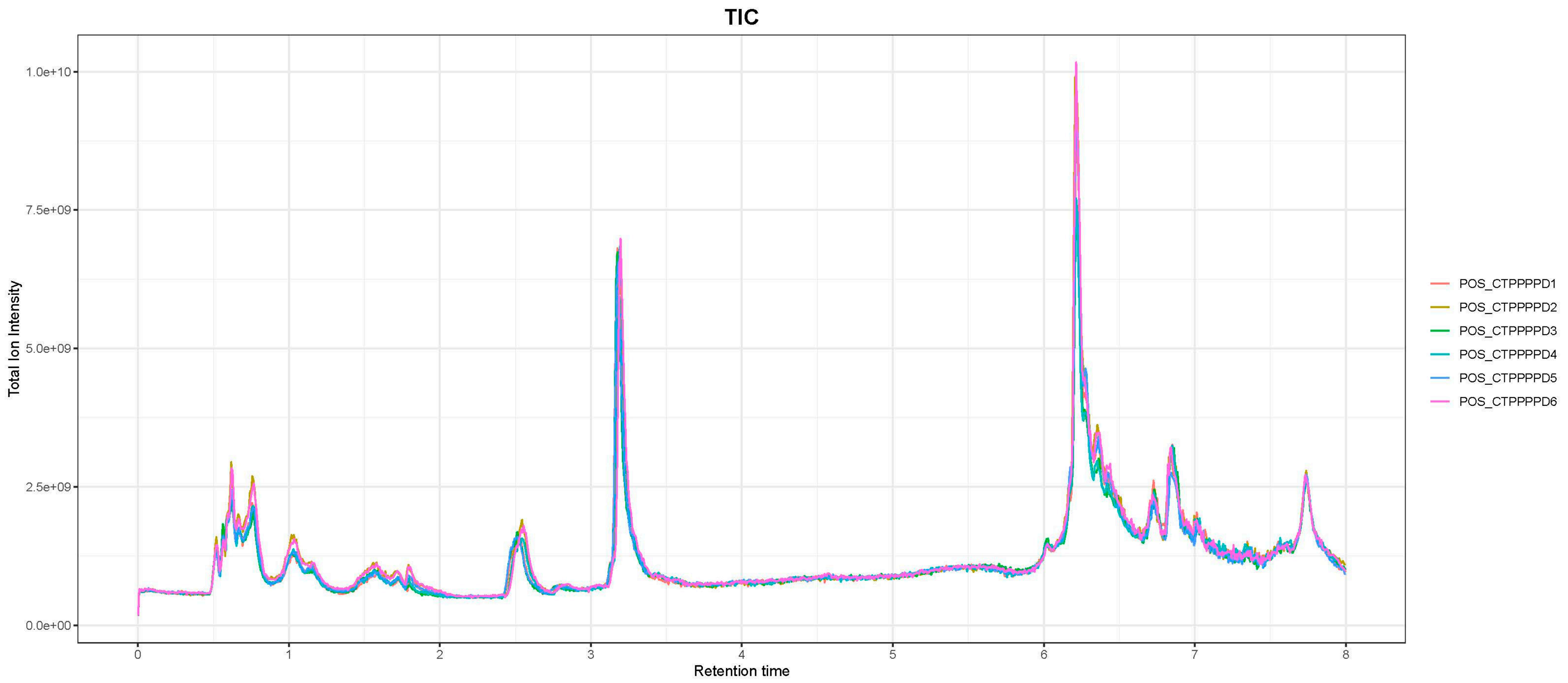

Figure S5. The total ion chromatogram in positive ion mode of compounds of CTPPPPD.

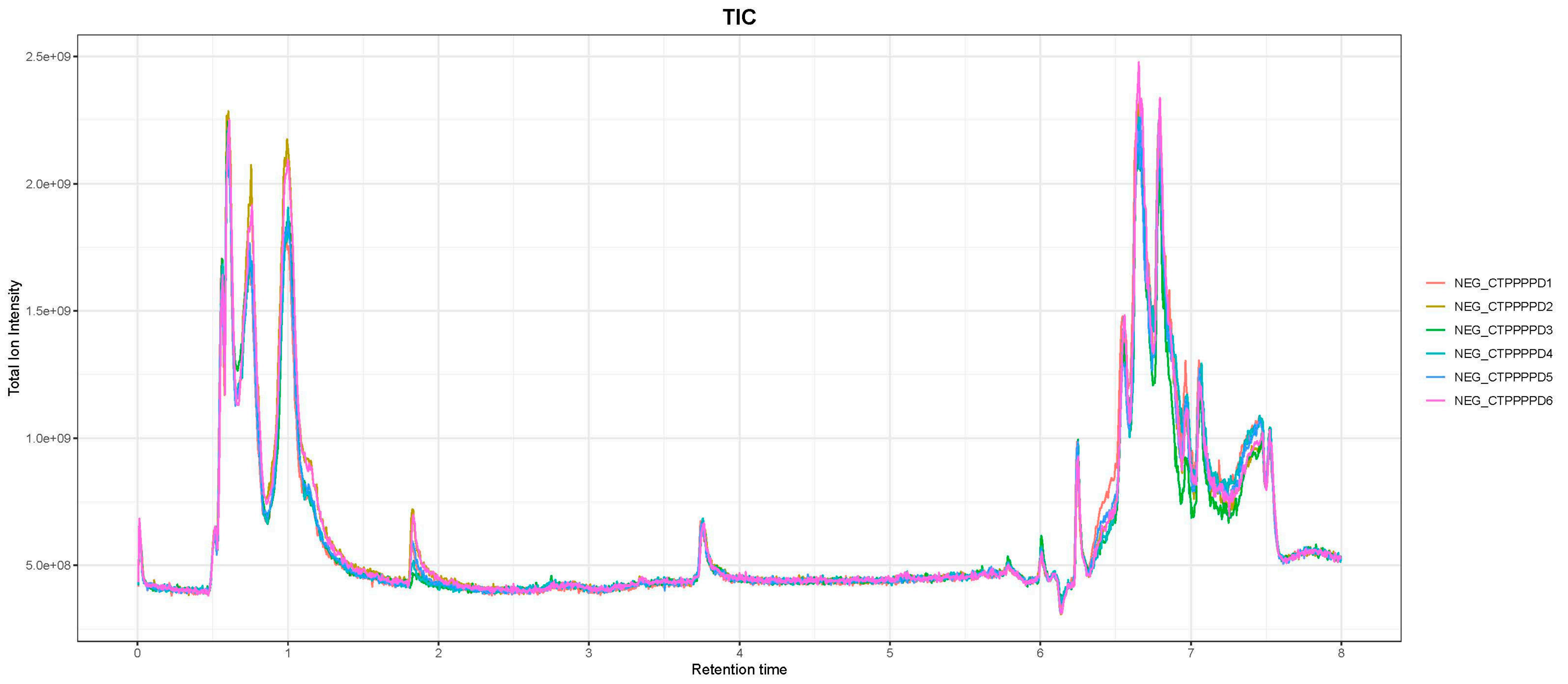

Figure S6. The total ion chromatogram in negative ion mode of compounds of CTPPPPD.

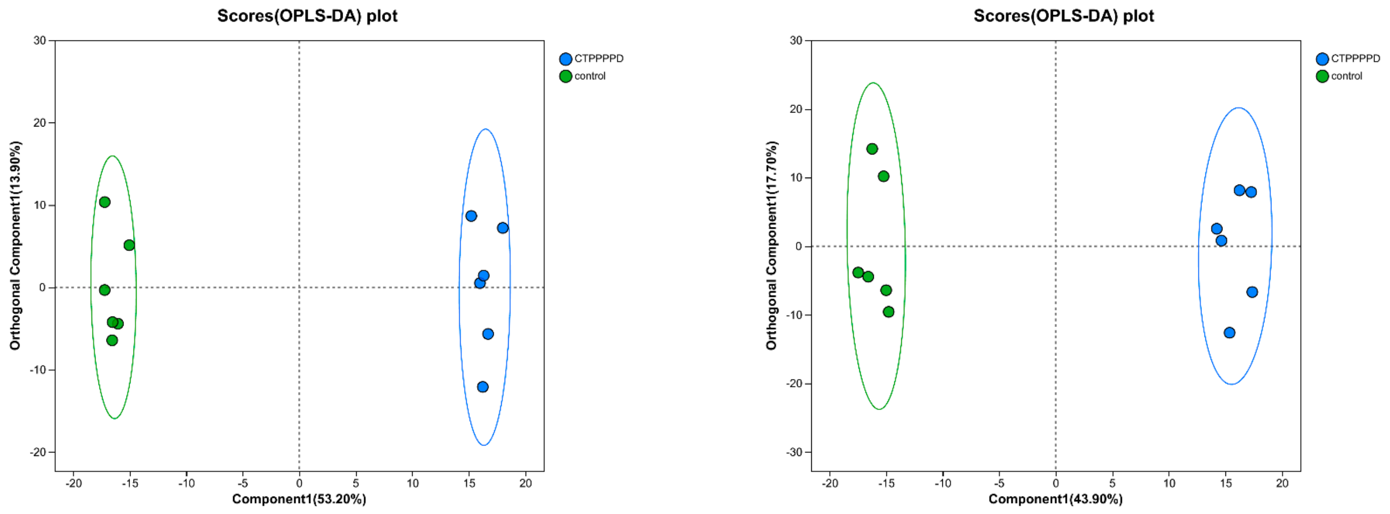

Figure S7. OPLS-DA score plot for the CTPPPPD and Control groups in positive ion mode and negative ion mode respectively.

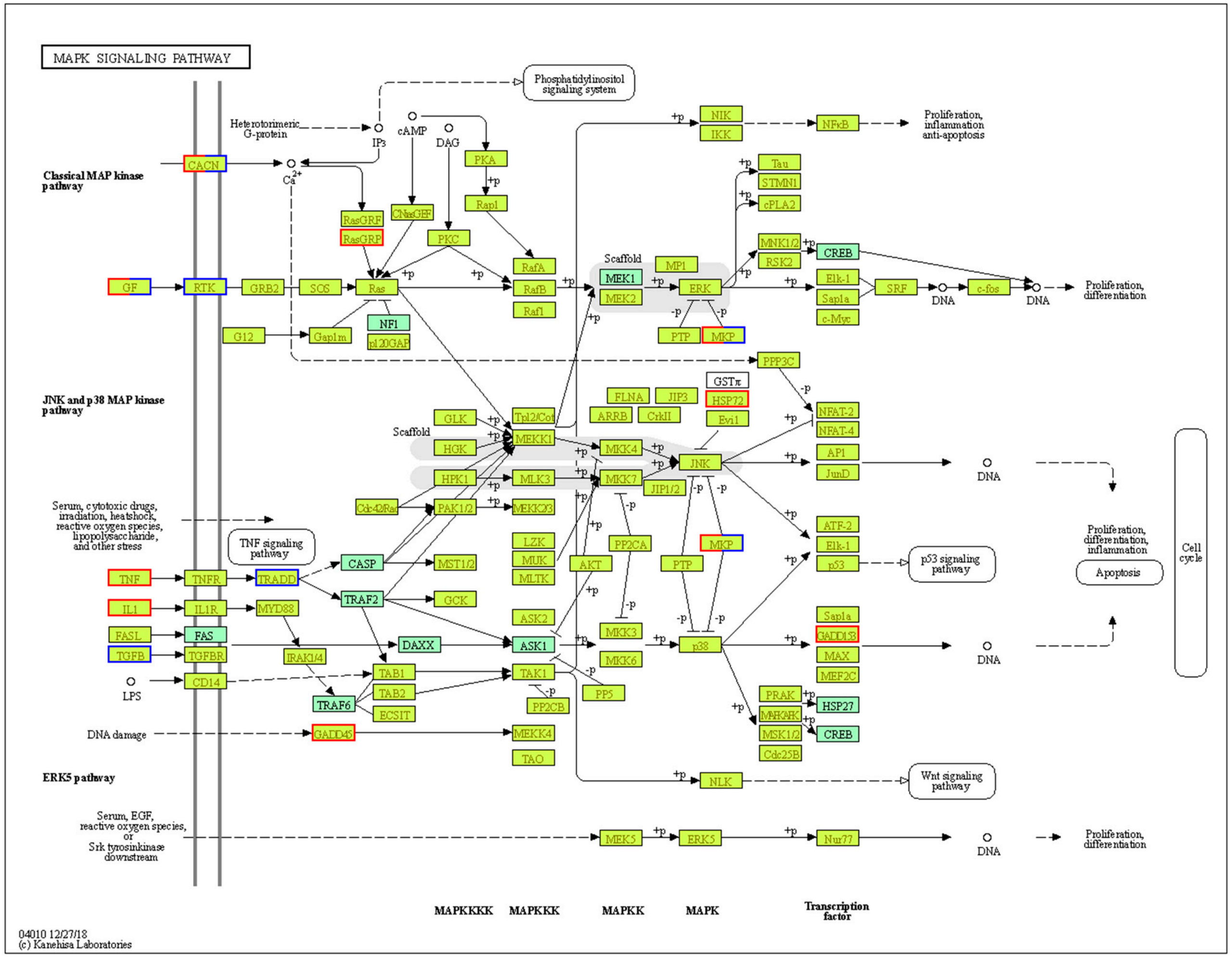

Figure S8. Enrichment of DEGs in MAPK signaling pathway.

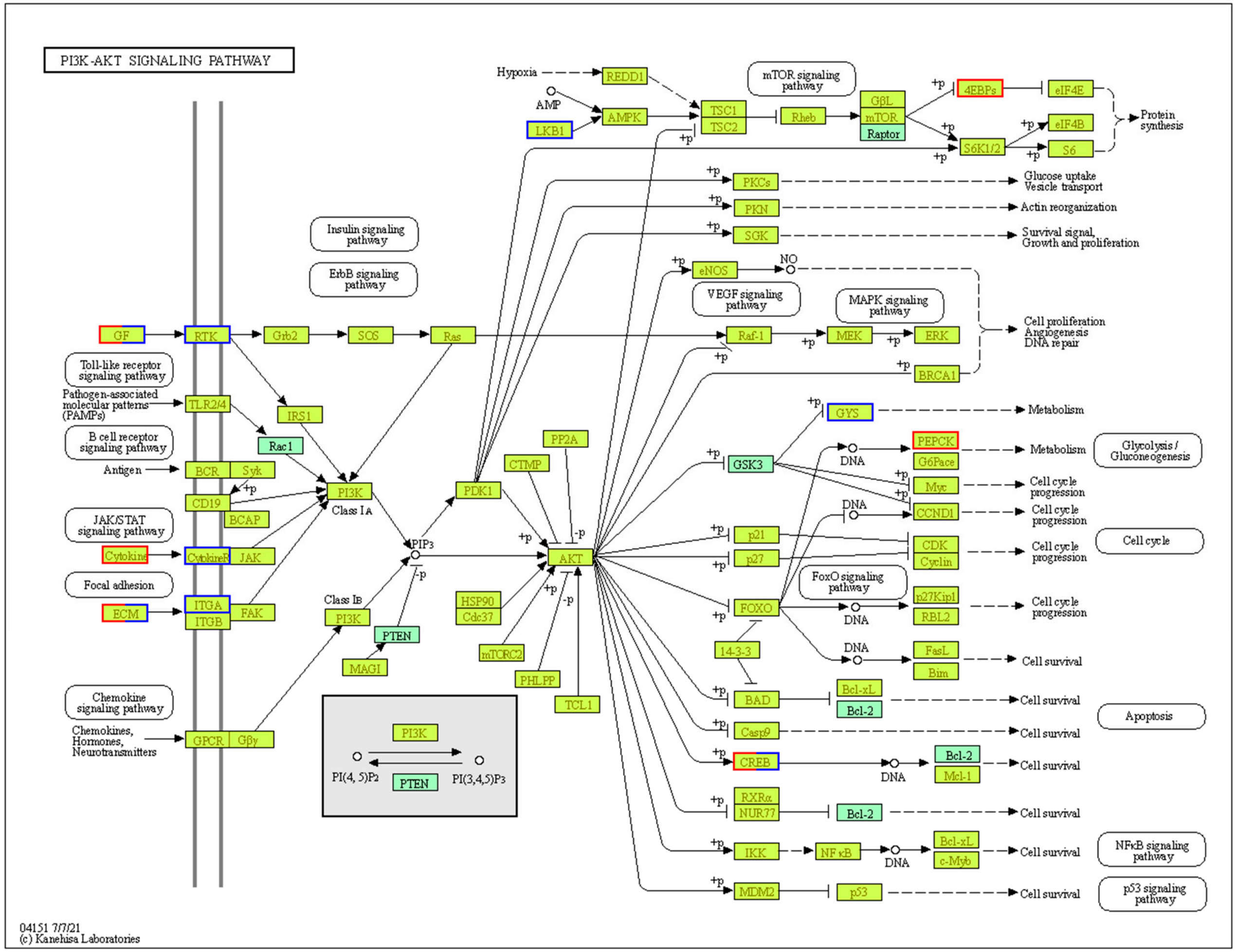

Figure S9. Enrichment of DEGs in PI3K-AKT signaling pathway.

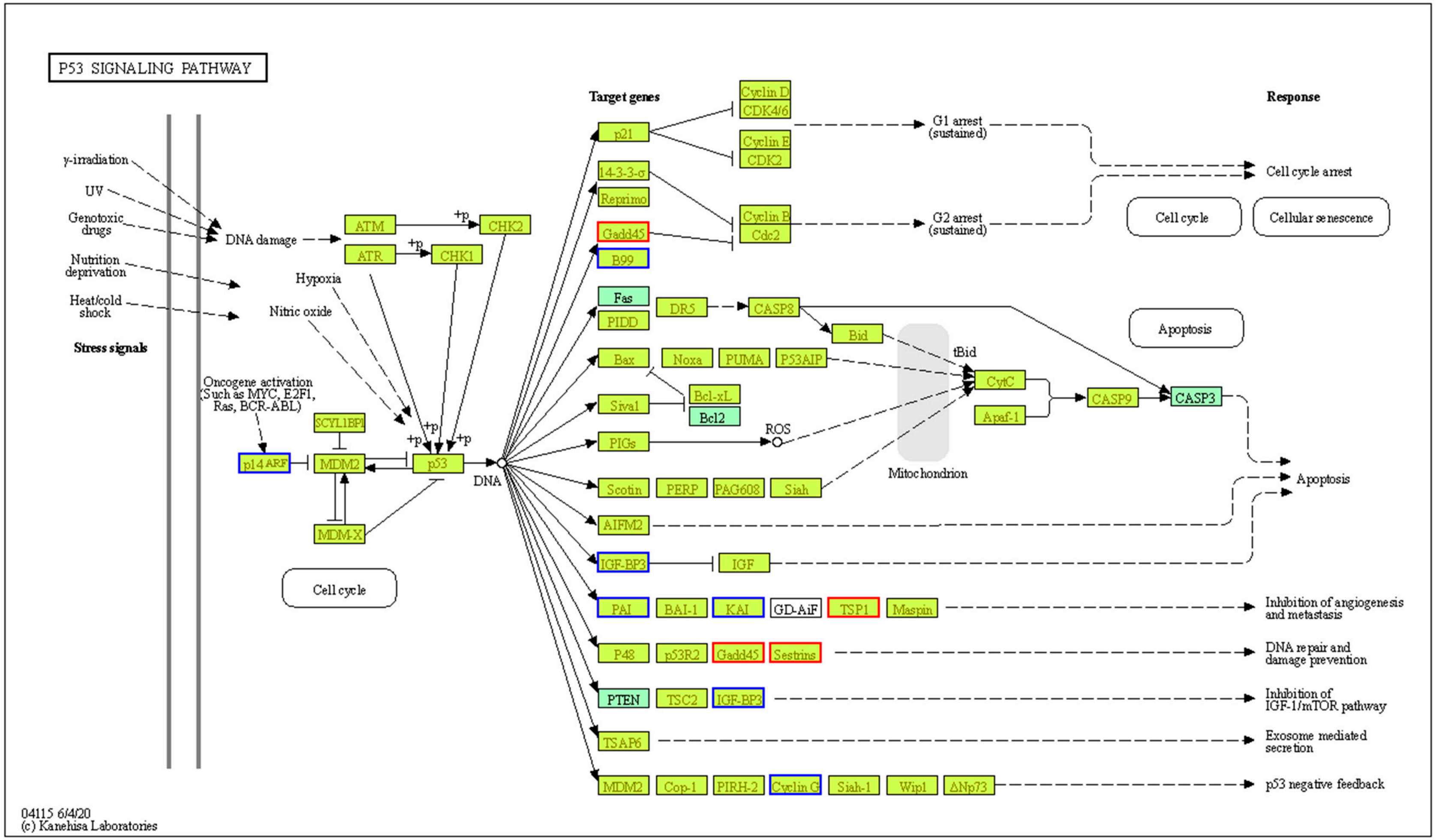

Figure S10. Enrichment of DEGs in P53 signaling pathway.

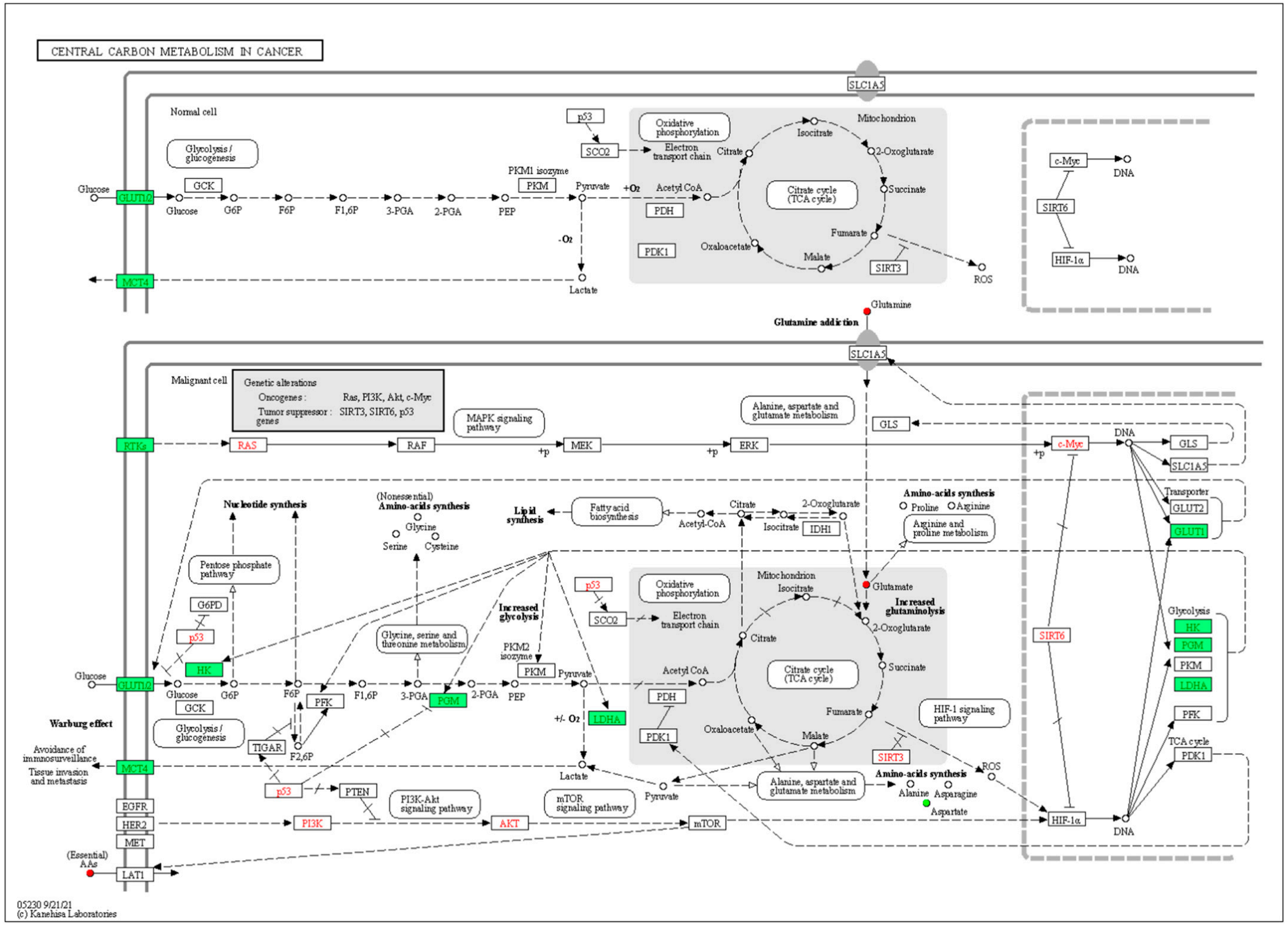

Figure S11. Enrichment of DEGs and DAMs in central carbon metabolism in cancer signaling pathway.

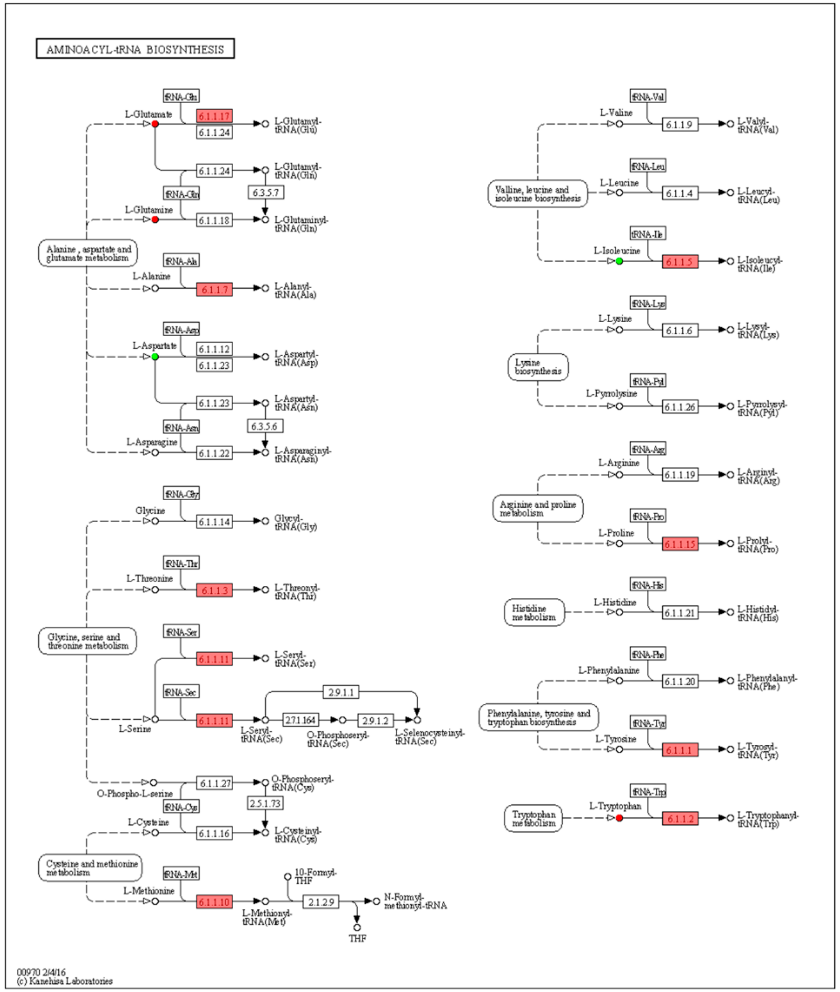

Figure S12. Enrichment of DEGs and DAMs in aminoacyl-tRNA biosynthesis signaling pathway.

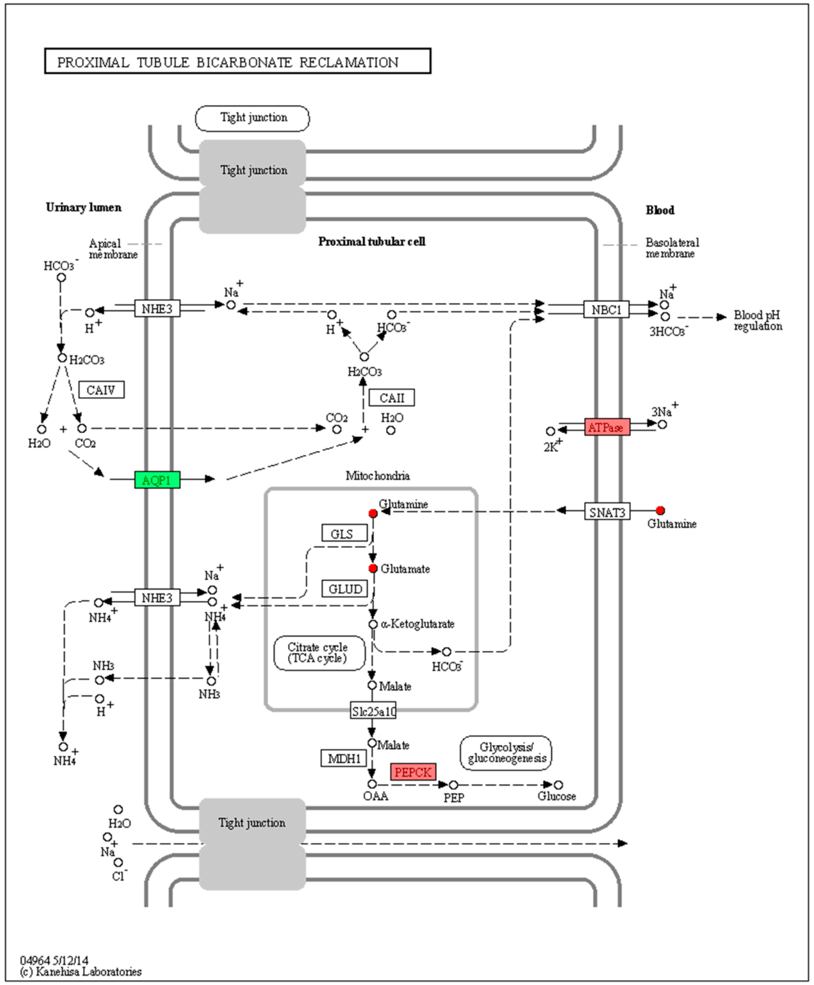

Figure S13. Enrichment of DEGs and DAMs in proximal tubule bicarbonate reclamation signaling pathway.

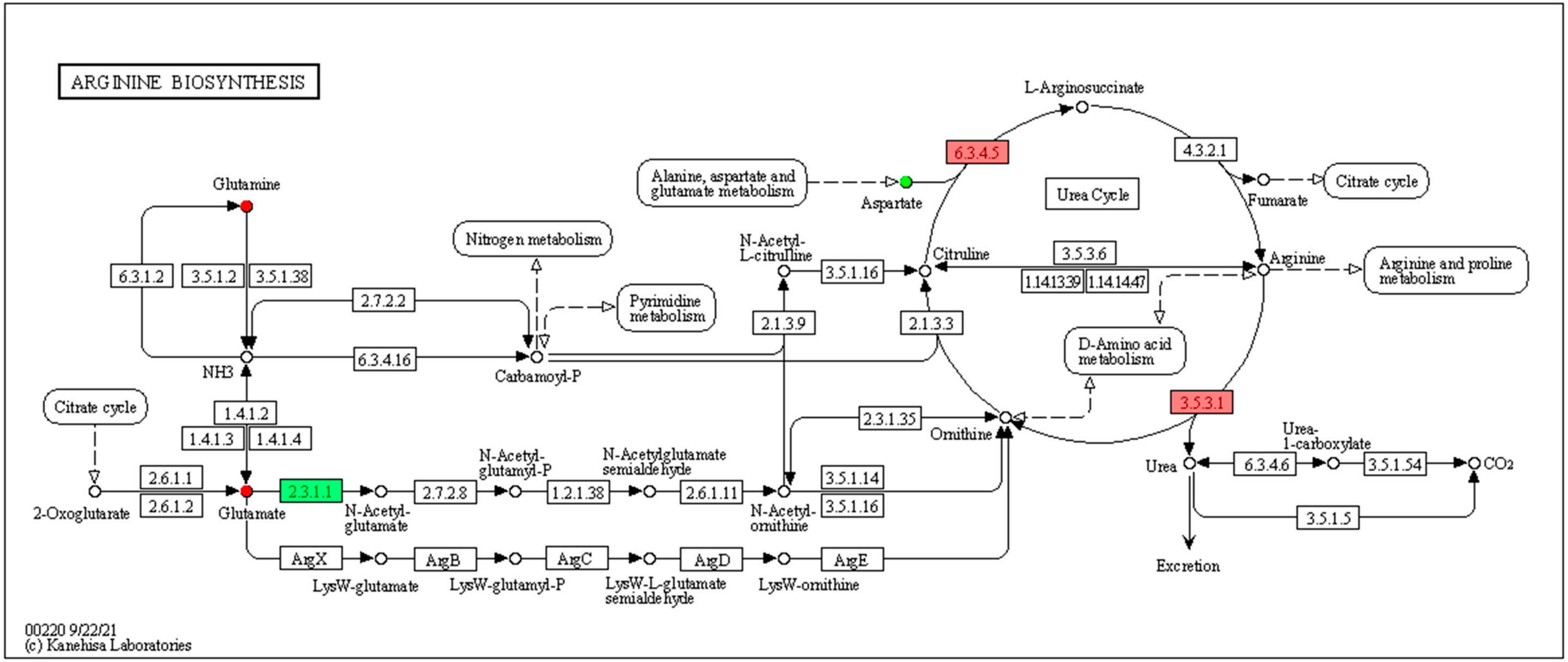

Figure S14. Enrichment of DEGs and DAMs in arginine biosynthesis signaling pathway.

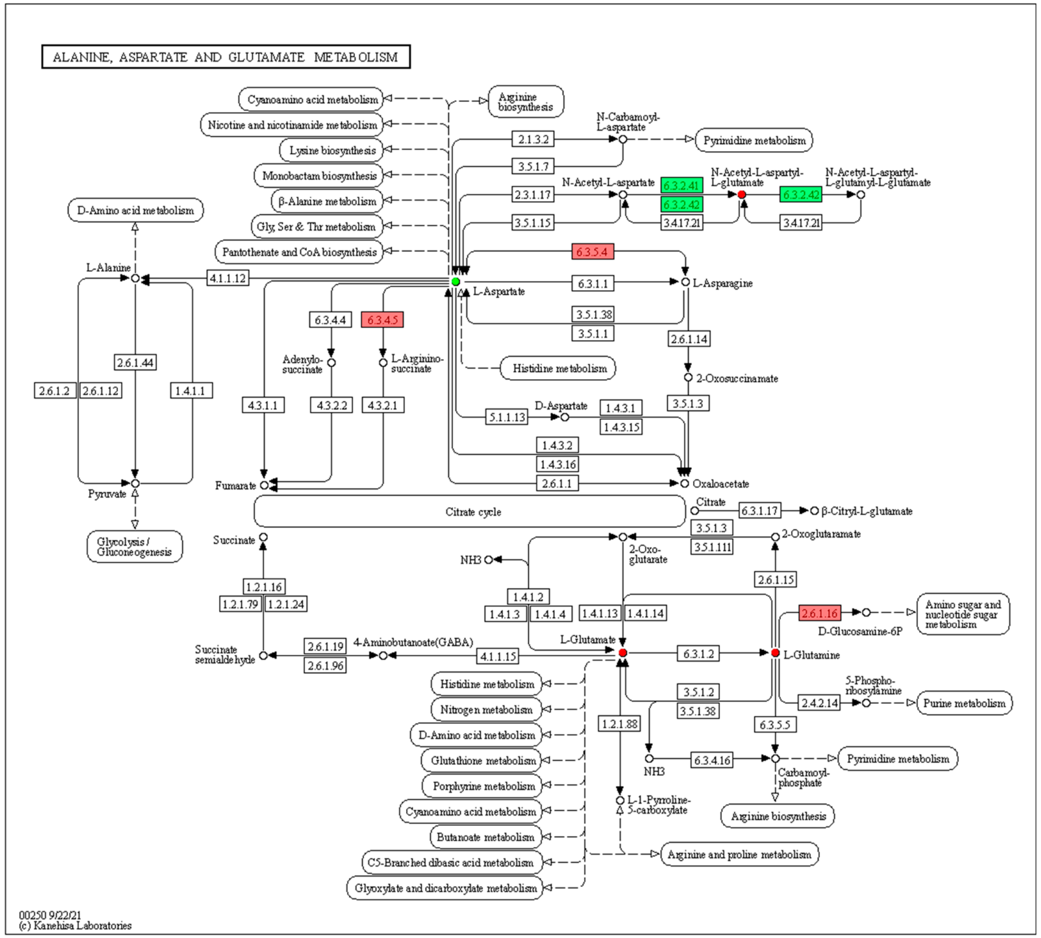

Figure S15. Enrichment of DEGs and DAMs in alanine, aspartate and glutamate metabolism signaling pathway
